# Glycosylation of plasma IgG in colorectal cancer prognosis

**DOI:** 10.1038/srep28098

**Published:** 2016-06-15

**Authors:** Evropi Theodoratou, Kujtim Thaçi, Felix Agakov, Maria N. Timofeeva, Jerko Štambuk, Maja Pučić-Baković, Frano Vučković, Peter Orchard, Anna Agakova, Farhat V. N. Din, Ewan Brown, Pauline M. Rudd, Susan M. Farrington, Malcolm G. Dunlop, Harry Campbell, Gordan Lauc

**Affiliations:** 1 The Usher Institute of Population Health Sciences and Informatics, University of Edinburgh, Teviot Place, Edinburgh, EH8 9AG, UK; 2 Colon Cancer Genetics Group, Institute of Genetics and Molecular Medicine, University of Edinburgh and Medical Research Council Human Genetics Unit, Crewe Road, Edinburgh, EH4 2XU, UK; 3 Genos Glycoscience Research Laboratory, Zagreb, Croatia, HR-10000; 4 Pharmatics Limited, Edinburgh Bioquarter, 9 Little France Road, Edinburgh, EH16 4UX, UK; 5 The Institute of Genetics and Molecular Medicine, Edinburgh Cancer Research Centre, University of Edinburgh, Western General Hospital, Crewe Road South, Edinburgh, EH4 2XR, UK; 6 National Institute for Bioprocessing Research & Training, Fosters Avenue, Mount Merrion, Blackrock, Co. Dublin, Ireland; 7 University of Zagreb Faculty of Pharmacy and Biochemistry, Zagreb, Croatia, HR-10000

## Abstract

In this study we demonstrate the potential value of Immunoglobulin G (IgG) glycosylation as a novel prognostic biomarker of colorectal cancer (CRC). We analysed plasma IgG glycans in 1229 CRC patients and correlated with survival outcomes. We assessed the predictive value of clinical algorithms and compared this to algorithms that also included glycan predictors. Decreased galactosylation, decreased sialylation (of fucosylated IgG glycan structures) and increased bisecting GlcNAc in IgG glycan structures were strongly associated with all-cause (q < 0.01) and CRC mortality (q = 0.04 for galactosylation and sialylation). Clinical algorithms showed good prediction of all-cause and CRC mortality (Harrell’s C: 0.73, 0.77; AUC: 0.75, 0.79, IDI: 0.02, 0.04 respectively). The inclusion of IgG glycan data did not lead to any statistically significant improvements overall, but it improved the prediction over clinical models for stage 4 patients with the shortest follow-up time until death, with the median gain in the test AUC of 0.08. These glycan differences are consistent with significantly increased IgG pro-inflammatory activity being associated with poorer CRC prognosis, especially in late stage CRC. In the absence of validated biomarkers to improve upon prognostic information from existing clinicopathological factors, the potential of these novel IgG glycan biomarkers merits further investigation.

Colorectal cancer (CRC) is the 4th most commonly diagnosed cancer in UK (13% of all cancers) and the 2nd most common cause of cancer death (10% of total) (Cancer Research UK). The risk of recurrence and death from CRC is related to tumour stage at diagnosis. The growing repertoire of treatments available for CRC, including new chemotherapy approaches, combined with challenging benefit:toxicity ratios and cost, highlights the importance of targeting interventions to patients most likely to benefit. Whilst clinico-pathological staging can stratify prognostic groups, it is limited in the precision with which it categorise poor/good prognosis tumours and informs treatment decisions at the individual level. This is clinically important, since patients with AJCC stage 2 CRC may be offered adjuvant chemotherapy if their cancer is classified as high risk^1^. In practice, pathological staging provides practically useful categorical classifications, however, stage 2 and 3 cancers comprise a spectrum of both apparent pathological features and also aggressiveness and the ability to subsequently metastasise. Furthermore, currently available tumour biomarkers assayed in blood perform poorly in terms of sensitivity, greatly limiting their value in cancer prognosis^2^. Hence, improving the discriminatory performance of pathological staging offers much potential for clinical benefit.

Human cells are covered with a layer of carbohydrates or glycans called the glycocalyx^3^. Glycosylation of proteins is an important post-translational modification for normal physiological processes such as protein folding, degradation and secretion and these changes are often instrumental in promoting cellular proliferation, inflammatory processes and metastasis^4^. There are several classes of glycans, including Asn (N)-linked and Ser/Thr (O)-linked glycans^3^. A number of different studies include preliminary reports of potentially important glycan biomarkers for cancer and other diseases^5^,^6^,^7^,^8^,^9^,^10^. However, technical challenges in analysing complex glycan structures have, thus far, hindered large scale investigation in human studies^4^,^11^,^12^. Many known cancer biomarkers are glycoproteins, but diagnostic tests often only measure the protein fraction, despite the fact that in many cases it has been convincingly demonstrated that assays of glycosylation status significantly improve diagnostic value of such biomarkers^13^,^14^.

Immunoglobulins (Igs) are glycoprotein molecules made by plasma cells in response to challenge from antigens such as those associated with microbiological agents or cancer cells and there have been previous reports that IgG antibodies can act as independent cancer prognostic factors^15^,^16^. Glycosylation is an important modulator of IgG function^17^,^18^. In this study, we explore the role of IgG glycosylation status as a novel prognostic biomarker of CRC, but also for classifying those patient groups with more aggressive tumours. This is the first large-scale investigation of the role of IgG *N*-glycans in cancer prognosis and is made possible by recent technological developments^19^.

We performed the first Genome Wide Association Study (GWAS) of the human IgG *N*-glycome and identified 9 single nucleotide polymorphisms (SNP) showing genome-wide association (p < 5 × 10^−8^)^20^. This included the *IKZF1* locus, which has been reported to be associated with the risk of various cancers^21^ and appears to be a key regulator of IgG core-fucosylation. In addition, in a parallel IgG *N*-glycome study we observed substantial inter-individual variation in IgG glycosylation. The levels of IgG molecules without core-fucose varied between 1.3% and 19% and we postulated that this may have a significant impact on antibody-dependent cellular cytotoxicity (ADCC) and thus capacity to eliminate cancer cells^22^.

We have recently reported that CRC associates with decrease in IgG galactosylation, IgG sialylation and increase in core-fucosylation of neutral glycans with concurrent decrease of core-fucosylation of sialylated glycans^23^. To examine the potential role of individual variation in IgG glycosylation on CRC prognosis we performed detailed characterisation of IgG glycome composition in 1229 CRC patients. In addition, we explored the prognostic biomarker potential of IgG glycans after stage stratification to account for the different stage prognosis of CRC patients.

## Results

### IgG glycan measurements

**Figure 1:**
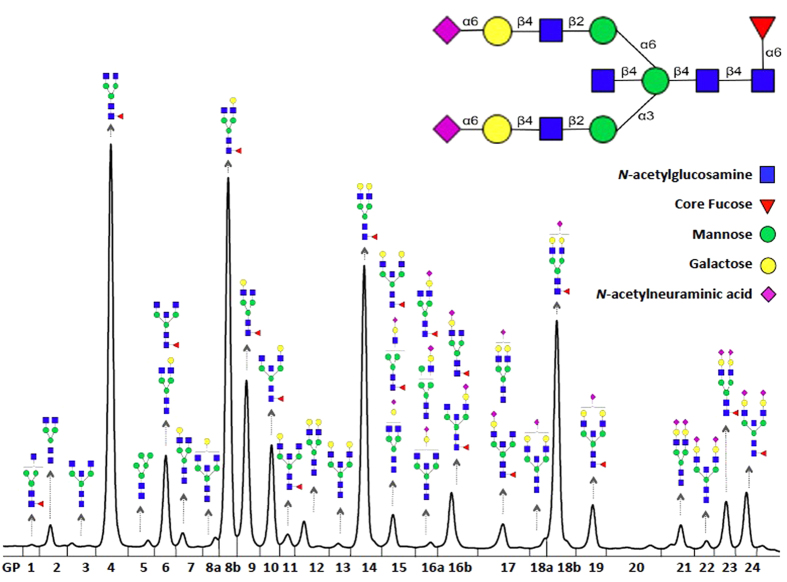
UPLC analysis of immunoglobulin G (IgG) glycosylation. Each IgG contains one conserved N-glycosylation site on Asn197 of its heavy chain. Different glycans can be attached to this site and the process seems to be highly regulated. UPLC analysis can reveal composition of the glycome attached to a population of IgG molecules by separating total IgG N-glycome into 24 chromatographic glycan peaks (GP1–GP24), mostly corresponding to individual glycan structures.

The IgG glycan analysis resulted in 23 directly measured IgG glycans structures, and 54 derived traits that represent common features shared among several measured glycans (galactosylation, sialylation, core fucosylation and the incidence of bisecting GlcNAc; Fig. 1, Supplementary Table 1)^22^,^24^. We restricted our survival analysis to those IgG glycan traits that were found to be robustly analysed. Robustness was calculated as follows. On each plate from the CRC cohort we put 3 standards that were biologically identical. Therefore, differences between measurements of standards are consequence of only experimental noise. We then calculated the variance of standards only and the variance in the whole CRC population. “Robustness” is defined as the ratio of those two variances (Var(Stand)/Var(CRC)) * 100 (i.e. lower values indicate higher robustness) and represents the contribution of experimental variation in total variation. Thirty nine of the 77 glycan traits whose percentage of experimental variation was below 20% were included in the analysis (Supplementary Table 1). The correlation coefficients of the robust measured and derived glycan traits are presented in Supplementary Fig. 1.

### Survival analysis

Among the 1229 patients, there were 9563 person-years of follow-up. There were 489 deaths, including 385 from CRC. Median follow-up was 9.4 years (IQR: 4.4 to 10.6 years) overall, and 10.3 years (IQR: 9.6 to 11.0 years) for live patients. Summary statistics and univariate Cox regression analysis for the confounding factors that were included in the subsequent glycan analysis are presented in Table 1. Of them stage at diagnosis and post-surgery CRP levels were strongly associated with all-cause and CRC-specific mortality (all-cause mortality: stage 3 vs. stage 1 OR (95% CI): 2.65 (1.96, 3.59), p-value 3.0 × 10^−10^; stage 4 vs. stage 1 OR (95% CI): 14.32 (10.37, 19.77), p-value 8.1 × 10^−19^; CRP levels >10 mg/l vs. ≤10 mg/l OR(95% CI): 2.13 (1.67, 2.72), p-value 1.1 × 10^−9^). Age at diagnosis, sex and site of cancer (colon or rectum) were not associated with all-cause or CRC-specific mortality.

**Figure 2:**
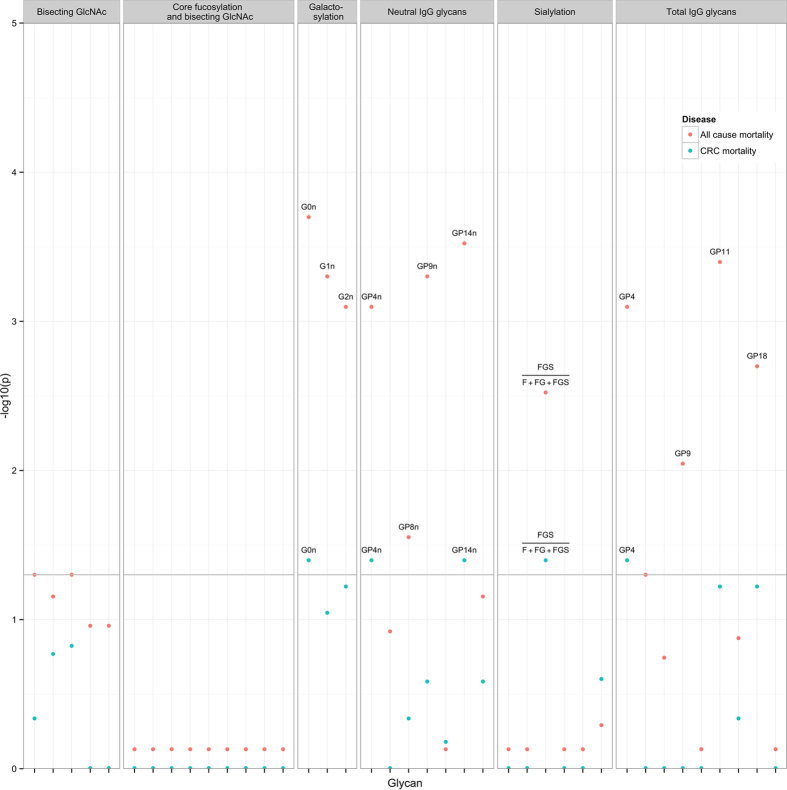
Manhattan-type plot of the association FDR corrected p values (q-values) of all 39 glycan variables for all–cause (red dots) and CRC-specific (blue dots) mortality adjusted for AJCC CRC stage, age, sex, time between sample and surgery, operation type, CRP and BMI (Model II). Analysed glycans are plotted on the X-axis. Y-axis plots the – logarithm of the q values. The names of glycans, which associations achieved statistical significance after correction for multiple testing (q < 0.05), are named in the plot. Details of the association analysis results are presented in Table 2 and cartoon structures of the glycans are presented in Fig. 1.

The univariate glycan HRs for the whole sample complete case analysis are presented in Table 2 and Supplementary Table 2 for all-cause mortality and in Table 2 and Supplementary Table 3 for CRC specific mortality. IgG glycans linked to mainly galactosylation were strongly associated with all-cause mortality and CRC mortality. In particular an increase in the percentage of agalactosylated structures (G0^n^) and a decrease in mono- and di-galactosylated structures (G1^n^, G2^n^) was associated with poorer all-cause and CRC-specific mortality. Statistically significant associations were also observed for decreased sialylation and increase in the incidence of bisecting GlcNAc (Table 2). Results were similar when AJCC stage 4 patients were excluded from the analysis (Supplementary Tables 4 and 5). The minus logarithm of the q-values (FDR corrected p-values) of all 39 glycan traits for all-cause mortality and CRC-specific model III are presented in a Manhattan-like plot (Fig. 2).

Stratified analysis by stage for all-cause and CRC-specific mortality is presented in Supplementary Tables 6 and 7. An increase in the percentage of agalactosylated structures (G0^n^) and a decrease in mono- and di-galactosylated structures (G1^n^, G2^n^) was associated with poorer all-cause and CRC-specific mortality in stages 1, 2 and 3 (p-values from all-cause mortality models for G0^n^: stage 1: 0.05, stage 2: 0.009 and stage 3: 0.01) but not in stage 4 (p-value for G0^n^: 0.38). In contrast, decrease in sialylation and increase in incidence of bisecting GlcNAc were statistically significantly associated with all-cause and CRC-specific mortality only in stage 4 (p-values from all-cause mortality models for stage 4 for FGS/(FG + FGS): 0.003; FGS/(F + FG + FGS): 0.01; FG2S1/(FG2 + FG2S1 + FG2S2): 0.002; FBG2S1/(FBG2 + FBG2S1 + FBG2S2): 0.008; FBS1/FS1: 0.008). Finally, only in stage 2 disease IgG glycans linked to core fucosylation were associated with all-cause and CRC-specific mortality (Supplementary Tables 6 and 7).

Multivariate Cox regression clinical algorithms (including all the covariates of model III) showed good prediction of subsequent all cause (Harrell’s C = 0.73, AUC = 0.75, IDI = 0.02 [as compared to model II that included AJCC stage, age and sex]) and CRC-mortality (Harrell’s C = 0.77, AUC = 0.79, IDI = 0.04 [as compared to model II that included AJCC stage, age and sex]). Using glycans in addition to the clinical factors (that were selected by generalised boosted regression) did not lead to any statistically significant improvements for the whole sample analysis (Table 3) or after stage stratification (Supplementary Tables 8 and 9). This was reconfirmed by using Cox regression with L1 (LASSO) penalties on model parameters^25^, as there were no significant differences in the validation deviances of models with and without glycans both for the whole sample and stage-stratified designs. Similarly, predictions of the 5-year risk of death using the clinical factors stage, age, sex, BMI and CRP (e.g. AUC = 0.80, Positive Predictive Value [PPV or precision] = 0.80, using the Naïve Bayes classifier with a kernel density estimator for the marginal distributions) were not improved by the addition of glycans data to the clinical factors. We refer the reader to the Supplementary Tables 10 and 11 and the Supplementary Section on Model Comparison for discussions and explanations.

When we stratified by stage, adding glycans to the clinical variables improved the prediction results, did not change them, or made them worse, depending on the stage of cancer and on the chosen models. We tested whether independently of the choice of a model class, adding glycans to clinical covariates would improve predictions of a model of the same class estimated on independent test data using cross-validation. We performed two instances of the paired Wilcoxon sign-rank test comparing models with and without glycans, including all the considered models (W), or including only the models of disparate classes (Wd) as discussed in Methods. We showed that there was no significant improvement in the prediction of the rapid progressors using glycans (in addition to the clinical factors) for stage 2 (p_W_ ~ 0.99, p_Wd_ ~ 0.98) as measured by cumulative (merged) AUC on the validation data (Supplementary Table 12). Similarly, for stage 3 the impact of the glycans was not consistent across the models, varied depending on the modelling assumptions, and was not significant overall (p_W_ ~ 0.75, p_Wd_ ~ 0.58; Supplementary Table 13).

On the other hand, there was a significant improvement in the prediction of the rapid progressors using glycans for stage 4 (Supplementary Table 14), with p_W_ ~ 0.01, p_Wd_ ~ 0.04, leading to the median gain in the test AUC of 0.08. Importantly, the inclusion of glycans in the models consistently resulted in the improved quality of predictions across the range of the considered models, and independently of whether the restricted or extended sets of clinical variables were used in the adjustments. The results were qualitatively similar for multiple repetitions of 10-fold cross-validation with random partitions into non-overlapping test folds, and independently of whether 10-fold or two-fold cross-validation was used to estimate the AUC on test data for the considered models. The best extended clinical model had the test AUC of 0.58, with the PPV of 0.35. The best model augmented with unfiltered log-transformed glycans had the test AUC of 0.66, with the PPV of 0.62. (Note: we acknowledge that since the choice of these best models uses the validation data, new external validations are needed to confirm the differences).

## Discussion

In this study, we investigated the relationship between the IgG glycome composition in plasma from of CRC patients with survival outcomes. We applied univariate and multivariate statistical models to examine the associations between specific glycan changes and CRC-specific or all-cause mortality. IgG glycans linked to galactosylation, sialylation and bisecting GlcNAc were strongly associated with all-cause mortality and CRC mortality. Since the method used to analyse glycans normalises measured data to the total glycome, this effectively measures glycome content per molecule of IgG and is not sensitive to changes in total IgG concentration. Multivariate Cox regression clinical algorithms showed good prediction of outcome for all cause and CRC-mortality, but using glycans in addition to the clinical factors did not lead to any statistically significant improvements. However, when we investigated the prediction of rapid progressors within each AJCC stage, there was an improvement in the prediction of the rapid progressors using glycans for stage 4.

It is well established that glycosylation changes are involved in the aetiology of cancer, and specifically mark tumour proliferation and metastasis^26^. IgG is produced and secreted by CRC cells and the expression levels of CRC-tumour derived IgG correlated with clinical and pathological characteristics of the tumour (including stage)^27^. In particular it has been shown that expression of IgG was stronger in CRC tissues with TNM stage III–IV, than in those with TNM I–II. Similarly, in this study we observe different changes in IgG glycosylation status (levels of sialylation and incidence of bisecting GlcNAC) in late-stage disease and we see an improvement in the prediction algorithms using glycans in addition to clinical factors in AJCC stage 4.

IgG biological activity is influenced by its Fc-glycosylation. Each heavy chain of IgG carries a single covalently attached bi-antennary N-glycan at the highly conserved asparagine 297 residue in each of the CH2 domains of the Fc region of the molecule. The attached oligosaccharides are structurally important for the stability of the antibody and its effector functions^28^. In addition, 15–20% of normal IgG molecules also bear complex bi-antennary oligosaccharides attached to the variable regions of the light chain, heavy chain or both^18^,^29^.

Changes in IgG galactosylation, sialylation, bisecting GlcNaC and fucosylation have been previously reported in cancer studies (Supplementary Table 15). In particular, a decline in plasma IgG galactosylation has been observed in multiple myeloma, prostate, gastric, lung and ovarian cancers^30^,^31^,^32^,^33^,^34^,^35^,^36^,^37^. Previous studies investigated small sample sizes (<100 cancer cases). This is the first time that similar changes in IgG galactosylation have been shown to be associated with CRC prognosis in a study with >1000 CRC patients (Supplementary Table 15). It has been shown that the immune system can identify and destroy new tumour cells through cancer immunosurveillance, which functions as an important defence against cancer. A recent review on the natural innate and adaptive immunity to cancer has presented evidence from mouse models that B cells (which create and release IgG) are important in the surveillance of CRC^38^. Furthermore, it has been hypothesised that decreased IgG galactosylation leads to a greater pro-inflammatory antibody response^39^,^40^, which might influence cancer survival after diagnosis. Interestingly, galactosylation of IgG increases during pregnancy and reverts to normal levels after delivery, indicating that IgG glycome composition is prone to natural modifications^41^.

Similar to galactosylation, decreased sialylation of IgG also results in a pro-inflammatory IgG phenotype^40^. In this study we found that decreased sialylation was also linked to poorer prognosis, which replicated findings of two small studies on ovarian^37^ and gastric cancer^33^. However, there was conflicting evidence in a study of multiple myeloma^42^, where increased sialylation of IgG was linked to higher risk of multiple myeloma. Therefore, through both decreased galactosylation and decreased sialylation, IgG in CRC patients with poorer prognosis had significantly greater pro-inflammatory properties (decreased galactosylation and sialylation) than CRC patients with better prognosis. Furthermore elevated occurrence of bisecting GlcNAc and lack of core fucose results in increased ADCC activity. In our study we found higher occurrence of bisecting GlcNAc in CRC patients of poorer prognosis, but IgG core fucosylation changes were associated with all-cause or CRC-specific mortality only in stage 2 CRC patients.

To our knowledge, this is the largest study to date examining the complexities of IgG glycan structure in relation to CRC prognosis. It includes prospective CRC cases from almost all hospitals in Scotland therefore is broadly representative of the colorectal cancer population. Cases were recruited as soon as possible after diagnosis to limit survival bias among those recruited and maximize the person-years of follow up. In addition, data relevant to the survival analysis were obtained from the Scottish registries General Register Office and the Scottish Cancer Registry (which are known to have high levels of data quality and data completeness) after linkage of our participants with their databases using the Community Health Index number. In addition, care was taken to determine the AJCC stage by experienced study clinicians reviewing individual computerized tomography scans and staging and metastasis information for every case. Information on date of diagnosis, date of sample taken and date of operation were collected and used in the univariate and multivariate models. Finally, we applied the state-of-the-art technology to measure the IgG glycosylation status in collaboration with the most experienced glyco-analytical laboratories in Europe.

This study has some limitations. One single measurement of IgG glycosylation status was taken after colorectal cancer diagnosis. Protein glycosylation status changes through time and is affected by factors such as age, operation and obesity. We have adjusted our analyses for all these factors, but it remains possible that glycosylation changes observed at the time of diagnosis do not reflect the glycosylation status at the time of death. This might also explain why IgG glycosylation status had more predictive power for late stage disease (since measurements were taken close to the end-event). However, the aim of this current work was to identify glycosylation changes that occur at colorectal cancer diagnosis and also investigate whether a measurement at time of diagnosis can act as a prognosis biomarker and therefore any biases due to single measurements are not relevant for these hypotheses. The stratified analysis leads to a reduction in the sample size, which may adversely affect the quality of the constructed predictive models. Finally, since our analysis included CRC patients of Scottish origin only, it may be possible that these findings will not be applicable to other ethnic groups. However, we have recently analysed the IgG glycosylation in 3 independent cohorts of Systemic Lupus Erythematosus (SLE) patients of different ethnicity and we observed very similar changes in IgG glycome composition in African Caribbean, Han Chinese, and Latin American Mestizo SLE patients despite known differences in SLE manifestation in different ethnic groups^43^. Therefore, there is a good reason to believe that the IgG glycosylation changes we observed in relation to CRC prognosis would be relevant to other ethnic populations too.

Some of the classification methods considered in this paper can be improved for handling imbalanced distributions of class labels. We are currently developing such extensions based on the advancements in imbalanced classification^44^, with the specific focus on predicting the prognosis for patients diagnosed at stages 1 and 2. The predictions may potentially be improved further by adapting recent approaches based on interaction networks^45^ of glycans and clinical factors. The definition of rapid progressors may require a refinement, especially at the earlier stages of colon cancer. For example, at stage 1, rather than defining a rapid progressor to be someone who dies of CRC during the lowest tertile of the follow-up, it could be useful to consider the speed of progression to the more advanced stages. We are currently investigating a range of alternative definitions. However, the key finding of IgG gyclans as a predictor of rapid CRC progression at the later stages remains potentially of great importance and should ideally be replicated in an independent study population.

The plasma IgG glycan differences which we observed at the time of CRC diagnosis are consistent with significantly increased IgG pro-inflammatory activity being associated with poorer CRC prognosis, especially in late stage (stages 3 and 4) CRC. In the absence of validated biomarkers to improve upon prognostic information from existing clinicopathological factors the potential of these novel IgG glycan biomarkers merits further investigation. In particular, the improved predictive power in models including glycan factors in stage 4 patients is interesting. Currently, there are various strategies that are employed when using chemotherapy^46^ in stage 4 disease. Therefore having a novel biomarker or prediction model that could help select patient groups that may have a better prognosis and a more indolent disease course would be useful as these patients could perhaps be offered chemotherapy agents with lower toxicity when compared to a more aggressive combination strategy. Furthermore, there is a great interest in novel immunotherapies in cancer and therefore it will be of useful to identify a more ‘immunogenic’ tumour phenotype based on identified IgG glyco-markers with a particular response to immunotherapy. Certainly to date the most encouraging results for immunotherapies (including PD-1 inhibitors) have been in tumours such as melanoma that are thought to be highly immunogenic and there is further interest in investigating mismatch repair deficient colon cancers which are often associated histologically with a heavy immune infiltrate. Finally, recent studies^47^ demonstrated that IgG glycosylation changes are very dynamic and variable between individuals, thus longitudinal studies are needed to fully investigate the prognostic potential of IgG glycosylation changes in CRC.

## Methods

### Study population

The SOCCS study (1999–2006) is a case-control study designed to identify genetic and environmental factors associated with non-hereditary colorectal cancer risk and survival outcome. Approval for the study was obtained from the MultiCentre Research Ethics Committee for Scotland and Local Research Ethics committee, and all participants gave written informed consent. The study has been described in detail elsewhere^48^.

The present study comprises 1229 patients with pathologically confirmed colorectal adenocarcinoma, in whom we assayed IgG glycan levels after CRC diagnosis. Participants completed a detailed lifestyle questionnaire and a semi-quantitative food frequency and supplements questionnaire (http://www.foodfrequency.org). Blood was collected and transferred to the research centre within 72 h of sampling. Plasma was prepared by gentle ficoll hypaque gradient centrifugation of sodium EDTA tubes and 1.5 mL of each participant’s plasma was stored at −80 °C.

### IgG glycans measurement and normalisation

All methods for IgG glycans measurement and normalisation were carried out in accordance with the approved guidelines as described as described previously^24^.

*Purification of IgG*: The IgG was isolated from plasma samples using 96-well protein G monolithic plates (BIA Separations, Ajdovščina, Slovenia). Briefly, 50–90 μl of plasma was diluted 10× with 1× PBS, pH 7.4 and applied to the protein G plate. IgG was eluted with 0.1 M formic acid (v = 1 mL; Merck, Darmstadt, Germany) and neutralized with 1 M ammonium bicarbonate (Merck).

*Release and labelling of IgG glycans* was performed as described previously^22^. IgG was first denatured with the addition of 30 μL 1.33% SDS (w/v) (Invitrogen, Carlsbad, CA, USA) and 10 min incubation at 65 °C. Subsequently, 10 μL of 4% Igepal-CA630 (Sigma-Aldrich, St. Louis, MO, USA) and 1.25 mU of PNGase F (ProZyme, Hayward, CA, USA) in 10 μL 5× PBS were added to the samples and incubated overnight at 37 °C to release *N*-glyans. The released *N*-glycans were labelled with 2-aminobenzamide (2-AB). The labelling mixture was freshly prepared by dissolving 2-AB (Sigma-Aldrich) in DMSO (Sigma-Aldrich) and glacial acetic acid (Merck) mixture (85:15, v/v) to a final concentration of 48 mg/mL. A volume of 25 μL of labelling mixture was added to each *N*-glycan sample in the 96-well plate. Also, 25 μL of freshly prepared reducing agent solution (106.96 mg/ml 2-picoline borane (Sigma-Aldrich) was added and the plate was sealed using adhesive tape. Mixing was achieved by shaking for 10 min, followed by 2 hour incubation at 65 °C. Samples (in a volume of 100 μL) were brought to 80% ACN (v/v) by adding 400 μL of ACN (J.T. Baker, Phillipsburg, NJ, USA). Free label and reducing agent were removed from the samples using HILIC-SPE. An amount of 200 μL of 0.1 g/mL suspension of microcrystalline cellulose (Merck) in water was applied to each well of a 0.45 μm GHP filter plate (Pall Corporation, Ann Arbor, MI, USA). Solvent was removed by application of vacuum using a vacuum manifold (Millipore Corporation, Billerica, MA, USA). All wells were prewashed using 5× 200 μL water, followed by equilibration using 3× 200 μL acetonitrile/water (80:20, v/v). The samples were loaded to the wells. The wells were subsequently washed 7× using 200 μL acetonitrile/water (80:20, v/v). Glycans were eluted with 2× 100 μL of water and combined eluates were stored at −20 °C until usage.

*Hydrophilic Interaction Chromatography* (*HILIC*)*-UPLC*: Fluorescently labelled *N*-glycans were separated by HILIC on a Waters Acquity UPLC instrument (Milford, MA, USA) with fluorescence detector set with excitation and emission wavelengths of 330 and 420 nm, respectively. The instrument was under the control of Empower 2 software, build 2145 (Waters, Milford, MA, USA). Labelled N-glycans were separated on a Waters BEH Glycan chromatography column, 100× 2.1 mm i.d., 1.7 μm BEH particles, with 100 mM ammonium formate, pH 4.4, as solvent A and acetonitrile as solvent B. Separation method used linear gradient of 75–62% acetonitrile (v/v) at flow rate of 0.4 ml/min in a 25 min analytical run. Samples were maintained at 5 °C before injection, and the separation temperature was 60 °C. The system was calibrated using an external standard of hydrolyzed and 2-AB labelled glucose oligomers from which the retention times for the individual glycans were converted to glucose units. Data processing was performed using an automatic processing method with a traditional integration algorithm after which each chromatogram was manually corrected to maintain the same intervals of integration for all the samples. The chromatograms were all separated in the same manner into 24 peaks. In addition to 24 directly measured glycan structures, 53 derived traits were calculated as described previously^22^. These derived traits average particular glycosylation features (galactosylation, fucosylation, sialylation) across different individual glycan structures. Consequently, they are more closely related to individual enzymatic activities, and underlying genetic polymorphisms.

*Glycan normalisation*: Normalization of glycan measurements across samples, was performed by total area of chromatograms, where peak area of each glycan was divided by total area of corresponding chromatogram.

### Survival and risk related parameters

Mortality outcomes were ascertained through linkage with the National Records of Scotland. Primary cause of death (“CRC” or “other”) was assigned from death certificates separately by two researchers (concordance was >99%). Time to event was measured from the date of diagnosis. Survival follow-up was censored at the date of death or at January, 31 2013, for participants who were not known to have died. Clinicopathological staging data was collected where possible (e.g. TNM is not feasible in patients who did not undergo surgery). Clinical records were reviewed and tumour site and multiplicity were determined from clinical and pathological records. Pre-operative staging imaging was collected through participating centres. Using the collated pathology, imaging and clinical data, tumour stage was assigned according TNM staging system and mapped onto the American Joint Committee on Cancer (AJCC) tumour-node-metastasis system (AJCC 1–4).

Blood was collected after CRC diagnosis (and after surgery). Median time to sampling was 5.4 months after the diagnosis (interquartile range, IQR: 3.2 to 8.3 months). Since illness and treatment may acutely affect IgG glycan levels and confound the analysis, a variable describing time from operation to blood collection and a variable determining the type of operation were created.

### Statistical analysis

Data were analysed using STATA (version 12.0) and R. We initially examined the association between IgG glycan levels (continuous and rank transformed) and CRC/all-cause mortality using Cox proportional hazards models. The non-CRC cause of death was right censored in CRC related mortality analysis. Tests of the proportional hazards assumptions and linearity on the log hazard rate scale were performed prior the analysis. Deviation from the proportional hazard assumption was noted for stage of disease variable in the overall analysis. No violation of assumption for any of the covariates was observed after stage stratification. Three models were applied in the whole dataset, in a dataset excluding stage 4 disease (to evaluate the glycan associations among patients with no-metastatic disease – AJCC stages 1–3): a crude model (Model I), a model where hazard ratios (HR) were adjusted for age at diagnosis, sex and stage of disease (Model II) and a model where HRs were adjusted for age at diagnosis, sex, stage of disease, body mass index (BMI), time from operation to blood collection, type of operation and CRP (Model III). P-values were adjusted for multiple testing using false discovery rate method (Benjamini–Hochberg procedure). Then we investigated whether the profiles of biomarkers differed across the stages of CRC by performing AJCC stage stratification.

We estimated the predictive value of a clinical-only Cox-regression algorithm for model II (which included age, sex, disease stage) and model III (which was adjusted for age, sex, disease stage, BMI and CRP level), by calculating the Harrell’s C concordance coefficient, the time-dependant cumulative/dynamic Area under the ROC curve (AUC) as suggested by Chambless, L. E. and G. Diao^49^ and the Integrated Discrimination Index (IDI defined as a difference in discrimination slopes) and compared this to an algorithm that also included glycan predictors. We ran this analysis in the whole data set and after AJCC stage stratification. The glycan variables included in the final model were selected by applying generalised boosted regression, which orders the variables by their relative importance, in 1000 bootstrap samples^50^,^51^, over the 10 inner training folds, and forward selection of ranked glycans by applying log-likelihood ratio test. The predictive value of the models was evaluated on independent samples using 10-fold cross-validation for all models except for AJCC stage 1 strata, where cross-validation was not possible due to the small number of events.

Finally, we performed classification analyses to a) predict whether a patient will die of CRC within 5-years from the time of diagnosis and b) to predict the rapid progressors within each stage. The analysis of the rapid progressors was pre-planned and motivated by the successful development of molecular signatures of prognostic biomarkers in other areas^52^; our goal here was to evaluate the potential of igG glycosylation as prognostic markers for CRC. A rapid progressor was defined as someone who died of CRC and whose follow-up time was in the lower 1/3^rd^ of the patients to die of CRC in that stage of cancer, with the cut-off thresholds at 2.9, 2.4, and 1.3 years for stages 2–4 respectively. We applied several families of classification models (LASSO, nearest neighbours, sparse shrunken centroids (PAM), Support Vector Machines, naive Bayes, Decision Trees, and boosted stump classifiers), with and without stratification, with and without initial filtering on the training data, with and without log transformations of glycan expressions and clinical factors. The choice of the models was influenced by their popularity in biomarker studies, and their ability to address high-dimensional (large-p, small-n) problems via regularization or an explicit control for model complexity. More information about these estimators is presented in Supplementary Box 1, and the motivations for considering multiple classifiers for this problem are discussed in Supplementary Section on Model Comparison. All the results for this analysis were aggregated over 10 runs using 10-fold cross validation, where the validation folds were used neither for filtering nor for estimation of model parameters. Where necessary and appropriate, we also used 10 inner folds to estimate the stopping criteria or optimal value of hyperparameters (such as the regularization parameter for LASSO).

Then we estimated whether adding glycans to clinical covariates would improve the predictive performance of a model of the same class on independent test data; that is, we compared LASSO using clinical variables with LASSO using clinical variables and glycans, DTs using clinical variables with DTs using clinical variables and glycans, etc. This task is different from the association analysis, or from identifying specific glyco-clinical models outperforming a known baseline, where corrections for multiple tests are needed to control the probability of false discoveries. We applied the paired Wilcoxon sign-rank test comparing models with and without glycans, testing whether the difference in the cross-validated AUC of the clinical and glycol-clinical models is significantly different from zero. One limitation of this approach is the assumption of independence of the paired observations. (Whereas the choice of the models is independent by construction, it is difficult to ensure independence of the estimates of the performance on new, previously unused test data without constructing an extensive simulation study mimicking the joint distribution of the glycans and clinical variables, which goes beyond the scope of this paper). To address this limitation, we performed the test twice, first by considering all the models, and then by considering models of disparate classes, where we retained one model of each class and discarded SVM-linear, SVM-quadratic, and SVM-cubic differing in the degree of the polynomial used for the kernel construction. We performed the tests multiple times for different training/test fold partitions of the 10-fold and 2-fold cross-validation, and found that the results were qualitatively similar irrespectively of the specifics of cross-validation.

## Additional Information

**How to cite this article**: Theodoratou, E. *et al.* Glycosylation of plasma IgG in colorectal cancer prognosis. *Sci. Rep.* **6**, 28098; doi: 10.1038/srep28098 (2016).

## Supplementary Material

Supplementary Information

## Figures and Tables

**Table 1:** Summary statistics and univariate Cox regression for factors influencing all-cause and CRC mortality.

All-cause mortality	Deceased Cases N = 489	Survived/Censored cases N = 740	p-value	HR (95% CI)	p-value
Mean age (SD)	59.94 (10.15)	58.59 (9.87)	0.02	1.01 (1.00, 1.02)	0.09
Sex					
* Men*	287	416		1.00	
* Women*	202	324	0.39	0.96 (0.80, 1.15)	0.67
AJCC stage					
* 1*	54	195		1.00	
* 2*	115	306		1.35 (0.98, 1.87)	0.07
* 3*	186	227		2.65 (1.96, 3.59)	3.0 × 10^−10^
* 4*	134	12	<10^−5^	14.32 (10.37, 19.77)	8.3 × 10^−19^
Site					
* Colon*	263	430		1.00	
* Rectum*	223	304	0.12	1.14 (0.95, 1.36)	0.15
Mean BMI (SD)^a^	26.86 (4.77)	26.32 (4.09)	0.05	1.03 (1.01, 1.05)	0.02
Mean CRP (SD)	5.99 (14.24)	2.54 (8.88)	<10^−5^	1.02 (1.01, 1.02)	1.2 × 10^−11^
CRP					
≤*10* *mg/l*	412	687			
>*10* *mg/l*	77	53	<10^−5^	2.13 (1.67, 2.72)	1.1 × 10^−9^
**CRC mortality**	**Deceased Cases N = 385**	**Survived/Censored cases N = 844**	**p-value**	**HR (95% CI)**	**p-value**
Mean age (SD)	58.81 (10.23)	59.27 (9.89)	0.46	1.00 (0.99, 1.01)	0.48
Sex					
* Men*	212	491		1.00	
* Women*	173	353	0.31	1.12 (0.91, 1.36)	0.28
AJCC stage					
* 1*	22	227		1.00	
* 2*	76	345		2.17 (1.35, 3.49)	0.001
* 3*	159	254		5.40 (3.46, 8.43)	1.3 × 10^−13^
* 4*	128	18	<10^−5^	30.63 (19.38, 48.40)	1.2 × 10^−48^
Site					
* Colon*	205	488		1.00	
* Rectum*	178	349	0.12	1.16 (0.95, 1.42)	0.15
Mean BMI (SD)^a^	27.08 (4.88)	26.29 (4.12)	0.007	1.04 (1.01, 1.06)	0.002
Mean CRP (SD)	6.57 (15.24)	2.71 (8.98)	<10^−5^	1.02 (1.01, 1.03)	4.5×10^−12^
CRP					
≤*10 mg/l*	318	781		1.00	
>*10 mg/l*	67	63	<10^−5^	2.37 (1.82, 3.09)	1.4 × 10^−10^

^a^BMI available for 1057 CRC cases (415 all-cause deaths and 642 survived/censored).

**Table 2:** All cause and CRC-specific analysis for rank transformed glycans.

Glycan	ALL cause analysis					CRC-specific analysis				
	Dead	Survived				Dead	Survived			
	(N = 489)	(N = 740)	Model II (AJCC, age, sex, time between sample and surgery, operation type, BMI, CRP, n = 952)			(N = 385)	(N = 844)	Model II (AJCC, age, sex, time between sample and surgery, operation type, CRP, bmi, n = 971)		
	Mean (SD)	Mean (SD)	HR (95% CI)	p-value	q-value	Mean (SD)	Mean (SD)	HR (95% CI)	p-value	q-value
Total IgG glycans (neutral and charged); Measured										
GP4	26.39 (7.33)	23.87 (6.37)	1.28 (1.14, 1.43)	2.6 × 10^−5^	0.0008	26.20 (7.31)	24.27 (6.59)	1.23 (1.09, 1.40)	0.001	0.04
GP6	6.34 (1.88)	5.79 (1.57)	1.19 (1.06, 1.34)	0.002	0.05	6.23 (1.85)	5.91 (1.63)	1.10 (0.97, 1.25)	0.14	0.99
GP8	18.26 (2.21)	18.67 (1.81)	0.87 (0.78, 0.97)	0.01	0.18	18.33 (2.13)	18.59 (1.91)	0.92 (0.81, 1.04)	0.16	0.99
GP9	9.38 (1.36)	9.83 (1.35)	0.83 (0.75, 0.92)	0.0003	0.009	9.46 (1.35)	9.74 (1.38)	0.90 (0.80, 1.01)	0.06	0.99
GP10	5.45 (1.23)	5.48 (1.15)	0.93 (0.84, 1.04)	0.2	0.74	5.40 (1.19)	5.50 (1.18)	0.90 (0.80, 1.02)	0.11	0.99
GP11	10.19 (3.13)	11.44 (3.13)	0.77 (0.68, 0.86)	9.6×10^−6^	0.0004	10.30 (3.19)	11.24 (3.15)	0.81 (0.71, 0.92)	0.002	0.06
GP15	1.38 (0.41)	1.50 (0.43)	0.86 (0.77, 0.96)	0.007	0.133	1.38 (0.40)	1.49 (0.43)	0.86 (0.76, 0.98)	0.02	0.46
GP18	7.78 (2.29)	8.47 (2.34)	0.79 (0.71, 0.89)	4.9×10^−5^	0.002	7.87 (2.31)	8.35 (2.34)	0.82 (0.72, 0.93)	0.002	0.06
GP19	1.87 (0.38)	1.90 (0.39)	0.98 (0.89, 1.08)	0.7	0.74	1.87 (0.37)	1.90 (0.39)	1.00 (0.90, 1.12)	0.98	0.99
Sialylation; Derived										
FGS/(FG+FGS)	24.78 (3.20)	24.95 (3.10)	0.93 (0.84, 1.03)	0.18	0.74	24.84 (3.24)	24.90 (3.09)	0.90 (0.80, 1.01)	0.08	0.99
FBGS/(FBG+FBGS)	32.83 (6.17)	32.58 (6.34)	1.04 (0.94, 1.15)	0.47	0.74	32.93 (6.13)	32.56 (6.34)	1.05 (0.94, 1.19)	0.38	0.99
FGS/(F+FG+FGS)	16.35 (3.63)	17.35 (3.61)	0.81 (0.72, 0.90)	0.0001	0.003	16.47 (3.64)	17.17 (3.63)	0.82 (0.72, 0.92)	0.001	0.04
FBGS/(FB+FBG+FBGS)	21.21 (4.88)	21.82 (4.96)	0.95 (0.86, 1.05)	0.3	0.74	21.38 (4.93)	21.66 (4.94)	0.99 (0.88, 1.11)	0.8	0.99
FG2S1/(FG2+FG2S1+FG2S2)	40.18 (2.99)	39.51 (2.77)	1.06 (0.95, 1.18)	0.28	0.74	40.22 (3.05)	39.57 (2.77)	1.00 (0.89., 1.13)	0.99	0.99
FBG2S1/(FBG2+FBG2S1+FBG2S2)	37.02 (3.87)	36.41 (3.97)	1.12 (1.01, 1.24)	0.03	0.51	37.11 (3.80)	36.44 (3.99)	1.17 (1.04, 1.31)	0.009	0.25
Bisecting GlcNAc; Derived										
FBS^total^/FS^total^	0.30 (0.08)	0.28 (0.07)	1.19 (1.06, 1.33)	0.002	0.05	0.30 (0.07)	0.29 (0.08)	1.17 (1.03, 1.33)	0.02	0.46
FBS1/FS1	0.17 (0.05)	0.16 (0.05)	1.18 (1.06, 1.32)	0.003	0.07	0.17 (0.05)	0.16 (0.05)	1.19 (1.05, 1.35)	0.006	0.17
FBS1/(FS1+FBS1)	0.14 (0.04)	0.14 (0.03)	1.19 (1.06, 1.32)	0.002	0.05	0.14 (0.03)	0.14 (0.03)	1.19 (1.06, 1.35)	0.005	0.15
FBS2/FS2	1.35 (0.32)	1.27 (0.30)	1.17 (1.05, 1.31)	0.005	0.11	1.33 (0.32)	1.29 (0.30)	1.10 (0.98, 1.25)	0.12	0.99
FBS2/(FS2+FBS2)	0.56 (0.06)	0.55 (0.06)	1.17 (1.05, 1.30)	0.005	0.11	0.56 (0.06)	0.55 (0.06)	1.10 (0.97, 1.25)	0.13	0.99
Neutral IgG glycans; Measured										
GP4^n^	32.33 (8.02)	29.56 (7.02)	1.28 (1.14, 1.43)	2.6×10^−5^	0.0008	32.14 (8.00)	29.98 (7.24)	1.24 (1.09, 1.41)	0.001	0.04
GP6^n^	7.78 (2.07)	7.18 (1.76)	1.17 (1.05, 1.31)	0.006	0.12	7.65 (2.07)	7.31 (1.83)	1.08 (0.94, 1.22)	0.27	0.99
GP8^n^	22.59 (3.09)	23.32 (2.52)	0.83 (0.74, 0.92)	0.001	0.028	22.71 (3.01)	23.18 (2.66)	0.87 (0.77, 0.98)	0.02	0.46
GP9^n^	11.60 (1.81)	12.28 (1.75)	0.79 (0.71, 0.88)	1.5×10^−5^	0.0005	11.72 (1.81)	12.14 (1.79)	0.85 (0.76, 0.97)	0.01	0.26
GP10^n^	6.73 (1.54)	6.85 (1.46)	0.90 (0.81, 1.01)	0.06	0.74	6.68 (1.49)	6.85 (1.50)	0.88 (0.77, 0.99)	0.03	0.66
GP14^n^	12.70 (4.29)	14.39 (4.37)	0.76 (0.68, 0.86)	7.4×10^−6^	0.0003	12.86 (4.38)	14.11 (4.38)	0.80 (0.71, 0.92)	0.001	0.04
GP15^n^	1.72 (0.54)	1.89 (0.59)	0.85 (0.76, 0.95)	0.003	0.07	1.72 (0.53)	1.87 (0.59)	0.85 (0.75, 0.97)	0.01	0.26
Galactosylation; Derived										
G0^n^	41.15 (9.19)	37.69 (8.09)	1.31 (1.16, 1.47)	5.5×10^−6^	0.0002	40.83 (9.18)	38.26 (8.36)	1.24 (1.09, 1.41)	0.001	0.04
G1^n^	42.72 (4.71)	44.26 (3.56)	0.78 (0.69, 0.87)	1.3×10^−5^	0.0005	42.90 (4.63)	43.99 (3.83)	0.82 (0.72, 0.94)	0.003	0.09
G2^n^	15.65 (5.00)	17.61 (5.15)	0.78 (0.69, 0.87)	2.4×10^−5^	0.0008	15.79 (5.09)	17.30 (5.16)	0.81 (0.71, 0.93)	0.002	0.06
Core fucosylation and bisecting GlcNAc; Derived										
F^n^	79.47 (3.74)	79.77 (3.46)	0.96 (0.86, 1.07)	0.48	0.74	79.68 (3.70)	79.63 (3.52)	1.02 (0.90, 1.15)	0.77	0.99
FG0^n^/G0^n^	78.81 (4.29)	78.69 (4.11)	1.04 (0.93, 1.16)	0.46	0.74	79.01 (4.23)	78.62 (4.16)	1.10 (0.97, 1.24)	0.13	0.99
FG1^n^/G1^n^	80.03 (3.79)	80.45 (3.56)	0.94 (0.85, 1.05)	0.31	0.74	80.24 (3.73)	80.30 (3.62)	1.00 (0.89, 1.14)	0.94	0.99
FB^n^	17.29 (3.20)	16.97 (2.90)	1.02 (0.91, 1.14)	0.73	0.74	17.10 (3.15)	17.09 (2.97)	0.95 (0.84, 1.08)	0.44	0.99
FBG0^n^/G0^n^	19.15 (3.88)	19.30 (3.65)	0.94 (0.84, 1.05)	0.25	0.74	18.98 (3.81)	19.36 (3.70)	0.89 (0.79, 1.01)	0.07	0.99
FBG1^n^/G1^n^	18.20 (3.46)	17.82 (3.25)	1.04 (0.93, 1.16)	0.5	0.74	18.01 (3.39)	17.96 (3.32)	0.97 (0.86, 1.10)	0.64	0.99
FB^n^/F^n^	0.22 (0.05)	0.21 (0.05)	1.02 (0.92, 1.14)	0.68	0.74	0.21 (0.05)	0.21 (0.05)	0.95 (0.84, 1.08)	0.47	0.99
FB^n^/F^n total^	17.88 (3.38)	17.55 (3.07)	1.02 (0.92 , 1.14)	0.68	0.74	17.68 (3.33)	17.68 (3.14)	0.96 (0.85, 1.09)	0.5	0.99
F^n^/(B^n^ +FB^n^)	4.65 (1.09)	4.71 (1.01)	0.98 (0.88, 1.09)	0.74	0.74	4.71 (1.06)	4.68 (1.03)	1.05 (0.92, 1.18)	0.47	0.99

Q value represents the adjusted p-values using the false discovery rate method (Benjamini–Hochberg procedure).

**Table 3:** Multivariate Cox regression of the a) clinical parameters and b) clinical and glycan parameters.

Clinical algorithm	All-cause mortality		Clinical algorithm	CRC mortality	
	HR (95% CI)	p-value		HR (95% CI)	p-value
Age	1.03 (1.01–1.04)	9.2 × 10^−6^	Age	1.02 (1.00–1.03)	0.02
Sex	0.91 (0.74–1.12)	0.36	Sex	1.03 (0.82–1.29)	0.82
AJCC stage 2 vs 1	1.30 (0.92–1.83)	0.14	AJCC stage 2 vs 1	2.22 (1.33–3.72)	0.002
AJCC stage 3 vs 1	2.44 (1.77–3.38)	7.3 × 10^−8^	AJCC stage 3 vs 1	5.02 (3.09–8.15)	6.7 × 10^−11^
AJCC stage 4 vs 1	15.92 (11.16–22.70)	<2.0 × 10^−16^	AJCC stage 4 vs 1	34.05 (20.60–56.29)	<2.0 × 10^−16^
CRP	1.96 (1.47–2.62)	4.9 × 10^−6^	CRP	2.08 (1.52–2.86)	4.8 × 10^−6^
BMI	1.04 (1.01–1.06)	0.002	BMI	1.05 (1.02–1.08)	0.0001
*Harrell’s C*	*0.73*		*Harrell’s C*	*0.77*	
*IDI*^a^	*0.02*		*IDI*^a^	*0.04*	
*AUC*	0.74		*AUC*	0.79	
**Clinical/glycans algorithm**	**All-cause mortality**		**Clinical/glycans algorithm**	**CRC mortality**	
	HR (95% CI)	p-value		HR (95% CI)	p-value
Age	1.02 (1.01–1.03)	0.003	Age	1.01 (0.99–1.02)	0.28
Sex	0.91 (0.74–1.12)	0.36	Sex	1.04 (0.82–1.30)	0.77
AJCC stage 2 vs 1	1.33 (0.94–1.88)	0.11	AJCC stage 2 vs 1	2.24 (1.34–3.74)	0.002
AJCC stage 3 vs 1	2.49 (1.80–3.45)	4.1 × 10^−8^	AJCC stage 3 vs 1	5.01 (3.09–8.14)	7.1 × 10^−11^
AJCC stage 4 vs 1	15.72 (10.99–22.49)	<2.0 × 10^−16^	AJCC stage 4 vs 1	33.63 (20.18–56.04)	<2.0 × 10^−16^
CRP	1.66 (1.23–2.25)	0.001	CRP	1.84 (1.31–2.59)	0.0004
BMI	1.03 (1.01–1.06)	0.006	BMI	1.05 (1.02–1.07)	0.0007
IGP48	1.69 (0.64–4.47)	0.29	IGP29	0.92 (0.81–1.05)	0.21
IGP26	0.72 (0.55–0.95)	0.02	IGP13	0.85 (0.74–0.97)	0.01
IGP8	0.53 (0.21–1.30)	0.16	IGP8	0.91 (0.81–1.03)	0.14
*Harrell’s C*	*0.73*		*Harrell’s C*	*0.77*	
*IDI*^b^	*0.05*		*IDI*^b^	*0.03*	
*AUC*	0.75		*AUC*	0.79	

^a^The IDI was calculated based on the comparison of model II (adjusted for stage, sex and age) and the full clinical model III (adjusted for stage, age, sex, bmi and CRP – presented here).

^b^The IDI was calculated based on the comparison of the full clinical model III (adjusted for stage, age, sex, bmi and CRP) and the full clinical model III with the three top selected glycans.
