# Supplementary material for: Glycosylation of plasma IgG in colorectal cancer prognosis
Source: Sci Rep. 2016 Jun 15;6:28098. doi: 10.1038/srep28098 (PMC4908421; doi:10.1038/srep28098)
Supplement: Supplementary Information [file srep28098-s1.pdf]

SUPPLEMENTARY MATERIAL FOR

Glycosylation of plasma IgG in colorectal cancer prognosis

Authors: Evropi Theodoratou[1,2,*], Kujtim Thaçi[3,*], Felix Agakov[4], Maria Timofeeva[2], Jerko Štambuk[3], Maja Pučić-Baković[3], Frano Vučković[3], Peter Orchard[4], Anna Agakova[4], Farhat VN Din[2], Ewan Brown[5], Pauline Rudd[6], Susan M Farrington[2], Malcolm G Dunlop[2], Harry Campbell[1,2,*], Gordan Lauc[2,7,*]

1 Centre for Population Health Sciences, University of Edinburgh, Edinburgh, UK

2 Colon Cancer Genetics Group, Institute of Genetics and Molecular Medicine, University of Edinburgh and Medical Research Council Human Genetics Unit, Edinburgh, UK

3 Genos Glycoscience Research Laboratory, Zagreb, Croatia

4 Pharmatics Limited, Edinburgh, UK

5 Edinburgh Cancer Research Centre, University of Edinburgh, Edinburgh, UK

6 National institute for bioprocessing research & training, Dublin, Ireland

7 University of Zagreb Faculty of Pharmacy and Biochemistry, Zagreb, Croatia

* Equal contribution from these authors

Correspondence: Dr Evropi Theodoratou, Centre for Population Health Sciences, University of Edinburgh, Edinburgh, UK, e.theodoratou@ed.ac.uk

Supplementary Box 1 Information about the estimators used in the classification analysis

LASSO - sparse logisitic regression. This is a standard logistic regression model with a logit link function, and L_1 penalties on the weights. To set the penalty parameter, we run 10-fold cross-validation over a set of 10 penalties in the range 0.001 to 0.1. We choose the penalty resulting in the minimum-deviance model.

k-nearest neighbours - k nearest neighbours with k=1. For a new input, this classifier predicts the class of the nearest input in the training data, measured by Euclidean distance.

PAM - Prediction Analysis for Microarrays. This is the "nearest-shrunken-centroid" classifier. To set the shrinkage parameter, we run 10-fold cross-validation over 31 equally spaced values in the range 0 to 1.5. We choose the parameter that produces the lowest mean error rate.

Support Vector Machines – kernel-based maximum-margin classifiers. We consider linear, square, cubic, and squared exponential (Gaussian) kernel functions with fixed hyperparameters.

Naive Bayes – factorized class-conditional classifiers with normal or kernel density estimator-based marginal distributions. The KDE bandwidth is selected automatically.

Decision Trees – non-parametric tree classifiers, with the internal nodes corresponding to predictors, and leaves encoding classification labels. Binary trees with the Gini impurity splitting criterion were used.

Boosted stump classifiers – aggregations of multiple boosted one-node decision trees (stumps), where each later stump focuses on previously misclassified samples, using a version of Adaboost.

Supplementary Table 1 Glycans annotation and experimental variation for each glycan variable

GROUP	Code	GENOS Code	Description	Formula	Variation
Total IgG glycans (neutral + charged)	IGP1	GP1	*The percentage of FA1 glycan in total IgG glycans*	*GP1 / GP * 100*	34.35
	IGP2	GP2	*The percentage of A2 glycan in total IgG glycans*	*GP2 / GP * 100*	34.02
	IGP3	GP4	*The percentage of FA2 glycan in total IgG glycans*	*GP4 / GP * 100*	2.45
	IGP4	GP5	*The percentage of M5 glycan in total IgG glycans*	*GP5 / GP * 100*	32.90
	IGP5	GP6	*The percentage of FA2B glycan in total IgG glycans*	*GP6 / GP * 100*	1.78
	IGP6	GP7	*The percentage of A2G1 glycan in total IgG glycans*	*GP7 / GP * 100*	29.37
	IGP7	GP8	*The percentage of FA2[6]G1 glycan in total IgG glycans*	*GP8 / GP * 100*	10.82
	IGP8	GP9	*The percentage of FA2[3]G1 glycan in total IgG glycans*	*GP9 / GP * 100*	6.71
	IGP9	GP10	*The percentage of FA2[6]BG1 glycan in total IgG glycans*	*GP10 / GP * 100*	2.42
	IGP10	GP11	*The percentage of FA2[3]BG1 glycan in total IgG glycans*	*GP11 / GP * 100*	31.34
	IGP11	GP12	*The percentage of A2G2 glycan in total IgG glycans*	*GP12 / GP * 100*	28.10
	IGP12	GP13	*The percentage of A2BG2 glycan in total IgG glycans*	*GP13 / GP * 100*	95.50
	IGP13	GP14	*The percentage of FA2G2 glycan in total IgG glycans*	*GP14 / GP * 100*	1.05
	IGP14	GP15	*The percentage of FA2BG2 glycan in total IgG glycans*	*GP15 / GP * 100*	12.56
	IGP15	GP16	*The percentage of FA2G1S1 glycan in total IgG glycans*	*GP16 / GP * 100*	33.19
	IGP16	GP17	*The percentage of A2G2S1 glycan in total IgG glycans*	*GP17/ GP * 100*	112.42
	IGP17	GP18	*The percentage of FA2G2S1 glycan in total IgG glycans*	*GP18 / GP * 100*	2.19
	IGP18	GP19	*The percentage of FA2BG2S1 glycan in total IgG glycans*	*GP19 / GP * 100*	15.42
	IGP19	GP20	*Structure not determined*	*GP20 / GP * 100*	104.69
	IGP20	GP21	*The percentage of A2G2S2 glycan in total IgG glycans*	*GP21 / GP * 100*	49.60
	IGP21	GP22	*The percentage of A2BG2S2 glycan in total IgG glycans*	*GP22 / GP * 100*	96.00
	IGP22	GP23	*The percentage of FA2G2S2 glycan in total IgG glycans*	*GP23 / GP * 100*	25.62
	IGP23	GP24	*The percentage of FA2BG2S2 glycan in total IgG glycans*	*GP24 / GP * 100*	30.39
Total IgG glycans -	IGP24	FGS/(FG+FGS)	*The percentage of sialylation of fucosylated galactosylated structures without bisecting GlcNAc in total IgG glycans*	*SUM(GP16 + GP18 + GP23) / SUM(GP16 + GP18 + GP23 + GP8 + GP9 + GP14) * 100*	18.10

derived parameters	IGP25	FBGS/(FBG+FBGS)	*The percentage of sialylation of fucosylated galactosylated structures with bisecting GlcNAc in total IgG glycans*	*SUM(GP19 + GP24) / SUM(GP19 + GP24 + GP10 + GP11 + GP15) * 100*	13.28
	IGP26	FGS/(F+FG+FGS)	*The percentage of sialylation of all fucosylated structures without bisecting GlcNAc in total IgG glycans*	*SUM(GP16 + GP18 + GP23) / SUM(GP16 + GP18 + GP23 + GP4 + GP8 + GP9 + GP14) * 100*	8.10
	IGP27	FBGS/(FB+FBG+FBGS)	*The percentage of sialylation of all fucosylated structures with bisecting GlcNAc in total IgG glycans*	*SUM(GP19 + GP24) / SUM(GP19 + GP24 + GP6 + GP10 + GP11 + GP15) * 100*	13.02
	IGP28	FG1S1/(FG1+FG1S1)	*The percentage of monosialylation of fucosylated monogalactosylated structures without bisecting GlcNAc in total IgG glycans*	*GP16 / SUM(GP16 + GP8 + GP9) * 100*	48.95
	IGP29	FG2S1/(FG2+FG2S1+FG2S2)	*The percentage of monosialylation of fucosylated digalactosylated structures without bisecting GlcNAc in total IgG glycans*	*GP18 / SUM(GP18 + GP14 + GP23) * 100*	9.74
	IGP30	FG2S2/(FG2+FG2S1+FG2S2)	*The percentage of disialylation of fucosylated digalactosylated structures without bisecting GlcNAc in total IgG glycans*	*GP23 / SUM(GP23 + GP14 + GP18) * 100*	22.02
	IGP31	FBG2S1/(FBG2+FBG2S1+FBG2S2)	*The percentage of monosialylation of fucosylated digalactosylated structures with bisecting GlcNAc in total IgG glycans*	*GP19 / SUM(GP19 + GP15 + GP24) * 100*	9.54
	IGP32	FBG2S2/(FBG2+FBG2S1+FBG2S2)	*The percentage of disialylation of fucosylated digalactosylated structures with bisecting GlcNAc in total IgG glycans*	*GP24 / SUM(GP24 + GP15 + GP19) * 100*	44.66
	IGP33	$F^{total}S1/F^{total}S2$	*Ratio of all fucosylated monosialylated and disialylated structures (+/- bisecting GlyNAc) in total IgG glycans*	*SUM(GP16 + GP18 + GP19) / SUM(GP23 + GP24)*	34.85
	IGP34	FS1/FS2	*Ratio of fucosylated monosialylated and disialylated structures (without bisecting GlcNAc) in total IgG glycans*	*SUM(GP16 + GP18) / GP23*	29.15
	IGP35	FBS1/FBS2	*Ratio of fucosylated monosialylated and disialylated structures (with bisecting GlcNAc) in total IgG glycans*	*GP19 / GP24*	45.81
	IGP36	FBS^{total}/FS^{total}	*Ratio of all fucosylated sialylated structures with and without bisecting GlcNAc in total IgG glycans*	*SUM(GP19 + GP24) / SUM(GP16 + GP18 + GP23)*	6.69
	IGP37	FBS1/FS1	*Ratio of fucosylated monosialylated structures with and without bisecting GlcNAc in total IgG glycans*	*GP19 / SUM(GP16 + GP18)*	5.67

	IGP38	FBS1/(FS1+FBS1)	*The incidence of bisecting GlcNAc in all fucosylated monosialylated structures in total IgG glycans in total IgG glycans*	*GP19 / SUM(GP16 + GP18 + GP19)*	5.48
	IGP39	FBS2/FS2	*Ratio of fucosylated disialylated structures with and without bisecting GlcNAc in total IgG glycans*	*GP24 / GP23*	3.89
	IGP40	FBS2/(FS2+FBS2)	*The incidence of bisecting GlcNAc in all fucosylated disialylated structures in total IgG glycans*	*GP24 / SUM(GP23 + GP24)*	4.44
Neutral IgG glycans	IGP41	$GP1^n$	*The percentage of FA1 glycan in total neutral IgG glycans (GP^n)*	*$GP1 / GP^n * 100$*	35.92
	IGP42	$GP2^n$	*The percentage of A2 glycan in total neutral IgG glycans (GP^n)*	*$GP2 / GP^n * 100$*	37.40
	IGP43	$GP4^n$	*The percentage of FA2 glycan in total neutral IgG glycans (GP^n)*	*$GP4 / GP^n * 100$*	1.27
	IGP44	$GP5^n$	*The percentage of M5 glycan in total neutral IgG glycans (GP^n)*	*$GP5 / GP^n * 100$*	36.24
	IGP45	$GP6^n$	*The percentage of FA2B glycan in total neutral IgG glycans (GP^n)*	*$GP6 / GP^n * 100$*	1.02
	IGP46	$GP7^n$	*The percentage of A2G1 glycan in total neutral IgG glycans (GP^n)*	*$GP7 / GP^n * 100$*	32.32
	IGP47	$GP8^n$	*The percentage of FA2[6]G1 glycan in total neutral IgG glycans (GP^n)*	*$GP8 / GP^n * 100$*	2.69
	IGP48	$GP9^n$	*The percentage of FA2[3]G1 glycan in total neutral IgG glycans (GP^n)*	*$GP9 / GP^n * 100$*	2.06
	IGP49	$GP10^n$	*The percentage of FA2[6]BG1 glycan in total neutral IgG glycans (GP^n)*	*$GP10 / GP^n * 100$*	0.91
	IGP50	$GP11^n$	*The percentage of FA2[3]BG1 glycan in total neutral IgG glycans (GP^n)*	*$GP11 / GP^n * 100$*	37.96
	IGP51	$GP12^n$	*The percentage of A2G2 glycan in total neutral IgG glycans (GP^n)*	*$GP12 / GP^n * 100$*	29.22
	IGP52	$GP13^n$	*The percentage of A2BG2 glycan in total neutral IgG glycans (GP^n)*	*$GP13 / GP^n * 100$*	92.53

	IGP53	$GP14^n$	*The percentage of FA2G2 glycan in total neutral IgG glycans (GP^n)*	*$GP14 / GP^n * 100$*	0.58
	IGP54	$GP15^n$	*The percentage of FA2BG2 glycan in total neutral IgG glycans (GP^n)*	*$GP15 / GP^n * 100$*	13.13
Neutral IgG glycans - derived parameters	IGP55	$G0^n$	*The percentage of agalactosylated structures in total neutral IgG glycans*	*$SUM(GP1^n: GP4^n + GP6^n)$*	1.24
	IGP56	$G1^n$	*The percentage of monogalactosylated structures in total neutral IgG glycans*	*$SUM(GP7^n: GP11^n)$*	1.09
	IGP57	$G2^n$	*The percentage of digalactosylated structures in total neutral IgG glycans*	*$SUM(GP12^n: GP15^n)$*	1.51
	IGP58	$F^{n\ total}$	*The percentage of all fucosylated structures (+/- bisecting GlcNAc) in total neutral IgG glycans*	*$SUM(GP1^n + GP4^n + GP6^n + GP8^n + GP9^n + GP10^n + GP11^n + GP14^n + GP15^n)$*	44.04
	IGP59	$FG0^{n\ total}/G0^n$	*The percentage of fucosylation of agalactosylated structures in total neutral IgG glycans*	*$SUM(GP1^n + GP4^n + GP6^n) / G0^n * 100$*	40.29
	IGP60	$FG1^{n\ total}/G1^n$	*The percentage of fucosylation of monogalactosylated structures in total neutral IgG glycans*	*$SUM(GP8^n + GP9^n + GP10^n + GP11^n) / G1^n * 100$*	20.74
	IGP61	$FG2^{n\ total}/G2^n$	*The percentage of fucosylation of digalactosylated structures in total neutral IgG glycans*	*$SUM(GP14^n + GP15) / G2^n * 100$*	81.14
	IGP62	F^n	*The percentage of fucosylated structures (without bisecting GlcNAc) in total neutral IgG glycans*	*$SUM(GP1^n + GP4^n + GP8^n + GP9^n + GP14^n)$*	11.33
	IGP63	$FG0^n/G0^n$	*The percentage of fucosylation of agalactosylated structures (without bisecting GlcNAc) in total neutral IgG glycans*	*$SUM(GP1^n + GP4^n) / G0^n * 100$*	4.70
	IGP64	$FG1^n/G1^n$	*The percentage of fucosylation of monogalactosylated structures (without bisecting GlcNAc) in total neutral IgG glycans*	*$SUM(GP8^n + GP9^n) / G1^n * 100$*	5.89
	IGP65	$FG2^n/G2^n$	*The percentage of fucosylation of digalactosylated structures (without bisecting GlcNAc) in total neutral IgG glycans*	*$GP14^n / G2^n * 100$*	57.82
	IGP66	FB^n	*The percentage of fucosylated structures (with bisecting GlcNAc) in total neutral IgG glycans*	*$SUM(GP6^n + GP10^n + GP11^n + GP15^n)$*	2.27
	IGP67	$FBG0^n/G0^n$	*The percentage of fucosylation of agalactosylated structures (with bisecting GlcNAc) in total neutral IgG*	*$GP6^n / G0^n * 100$*	1.06

			glycans		
	IGP68	FBG1^n/G1^n	*The percentage of fucosylation of monogalactosylated structures (with bisecting GlcNAc) in total neutral IgG glycans*	*SUM(GP10^n + GP11^n) / G1^n * 100*	2.70
	IGP69	FBG2^n/G2^n	*The percentage of fucosylation of digalactosylated structures (with bisecting GlcNAc) in total neutral IgG glycans*	*GP15^n / G2^n * 100*	21.03
	IGP70	FB^n/F^n	*Ratio of fucosylated structures with and without bisecting GlcNAc in total neutral IgG glycans*	*FB^n/ F^n * 100*	3.39
	IGP71	FB^n/F$^{n\ total}$	*The incidence of bisecting GlcNAc in all fucosylated structures in total neutral IgG glycans*	*FB^n/ F$^{n\ total}$ * 100*	3.43
	IGP72	F^n/(B^n + FB^n)	*Ratio of fucosylated non-bisecting GlcNAc structures and all structures with bisecting GlcNAc in total neutral IgG glycans*	*F^n/(GP13^n + FB^n)*	6.00
	IGP73	B^n/(F^n + FB^n)	*Ratio of structures with bisecting GlcNAc and all fucosylated structures (+/- bisecting GlcNAc) in total neutral IgG glycans*	*GP13^n/ (F^n+ FB^n) * 1000*	92.61
	IGP74	FBG2^n/FG2^n	*Ratio of fucosylated digalactosylated structures with and without bisecting GlcNAc in total neutral IgG glycans*	*GP15^n/GP14^n*	28.12
	IGP75	FBG2^n /(FG2^n + FBG2^n)	*The incidence of bisecting GlcNAc in all fucosylated digalactosylated structures in total neutral IgG glycans*	*GP15^n/(GP14^n + GP15^n) * 100*	28.43
	IGP76	FG2^n/(BG2^n + FBG2^n)	*Ratio of fucosylated digalactosylated non-bisecting GlcNAc structures and all digalactosylated structures with bisecting GlcNAc in total neutral IgG glycans*	*GP14^n/(GP13^n + GP15^n)*	52.69
	IGP77	BG2^n/(FG2^n + FBG2^n)	*Ratio of digalactosylated structures with bisecting GlcNAc and all fucosylated digalactosylated structures (+/- bisecting GlcNAc) in total neutral IgG glycans*	*GP13^n/(GP14^n + GP15^n) * 1000*	106.54

Supplementary Table 2 All cause analysis

Code	Glycan	Dead (N=489)	Survived (N=740)	Crude model (n=1229)		Model I (AJCC, age, sex, n=1229)		Model II (AJCC, age, sex, time between sample and surgery, operation type, bmi, CRP n=952)	
		Mean (SD)	Mean (SD)	HR (95% CI)	p-value	HR (95% CI)	p-value	HR (95% CI)	p-value
**Total IgG glycans (neutral and charged); Measured**									
IGP3	Continuous	26.39 (7.33)	23.87 (6.37)	1.04 (1.03, 1.06)	$4.7x10^{-12}$	1.04 (1.03, 1.06)	$7.5x10^{-11}$	1.04 (1.02, 1.05)	$7.5x10^{-6}$
	RT			1.36 (1.24, 1.48)	$5.9x10^{-11}$	1.35 (1.23, 1.48)	$5.0x10^{-10}$	1.28 (1.14, 1.43)	$2.6x10^{-5}$
IGP5	Continuous	6.34 (1.88)	5.79 (1.57)	1.15 (1.09, 1.21)	$1.5x10^{-8}$	1.14 (1.08, 1.20)	$3.5x10^{-7}$	1.11 (1.04, 1.18)	0.001
	RT			1.28 (1.17, 1.40)	$9.7x10^{-8}$	1.27 (1.15, 1.39)	$1.4x10^{-6}$	1.19 (1.06, 1.34)	0.002
IGP7	Continuous	18.26 (2.21)	18.67 (1.81)	0.91 (0.87, 0.96)	0.0001	0.93 (0.88, 0.97)	0.001	0.92 (0.87, 0.97)	0.003
	RT			0.85 (0.77, 0.93)	0.001	0.87 (0.79, 0.95)	0.003	0.87 (0.78, 0.97)	0.01
IGP8	Continuous	9.38 (1.36)	9.83 (1.35)	0.83 (0.78, 0.89)	$3.4x10^{-8}$	0.86 (0.80, 0.91)	$2.6x10^{-6}$	0.87 (0.81, 0.94)	0.0002
	RT			0.78 (0.74, 0.85)	$3.4x10^{-8}$	0.81 (0.74, 0.89)	$4.4x10^{-6}$	0.83 (0.75, 0.92)	0.0003
IGP9	Continuous	5.45 (1.23)	5.48 (1.15)	0.96 (0.89, 1.04)	0.35	0.96 (0.88, 1.03)	0.26	0.95 (0.87, 1.04)	0.29
	RT			0.95 (0.87, 1.04)	0.25	0.94 (0.85, 1.03)	0.16	0.93 (0.84, 1.04)	0.20
IGP13	Continuous	10.19 (3.13)	11.44 (3.13)	0.89 (0.87, 0.92)	$8.9x10^{-13}$	0.90 (0.87, 0.93)	$1.4x10^{-10}$	0.92 (0.89, 0.96)	$4.0x10^{-5}$
	RT			0.70 (0.64, 0.77)	$5.2x10^{-14}$	0.71 (0.65, 0.79)	$1.6x10^{-11}$	0.77 (0.68, 0.86)	$9.6x10^{-6}$
IGP14	Continuous	1.38 (0.41)	1.50 (0.43)	0.55 (0.44, 0.70)	$4.0x10^{-7}$	0.59 (0.46, 0.74)	$5.9x10^{-6}$	0.70 (0.53, 0.92)	0.01
	RT			0.78 (0.71, 0.85)	$9.0x10^{-8}$	0.80 (0.73, 0.88)	$2.0x10^{-6}$	0.86 (0.77, 0.96)	0.007
IGP17	Continuous	7.78 (2.29)	8.47 (2.34)	0.89 (0.85, 0.93)	$9.3x10^{-8}$	0.89 (0.85, 0.93)	$1.3x10^{-7}$	0.91 (0.86, 0.96)	0.0002
	RT			0.76 (0.70, 0.83)	$4.4x10^{-9}$	0.76 (0.69, 0.83)	$8.4x10^{-9}$	0.79 (0.71, 0.89)	$4.9x10^{-5}$
IGP18	Continuous	1.87 (0.38)	1.90 (0.39)	0.85 (0.67, 1.08)	0.18	0.81 (0.65, 1.02)	0.08	0.94 (0.73, 1.22)	0.64
	RT			0.94 (0.86, 1.03)	0.18	0.93 (0.85, 1.01)	0.10	0.98 (0.89, 1.08)	0.70
**Sialylation**									
IGP24	Continuous	24.78 (3.20)	24.95 (3.10)	0.99 (0.96, 1.01)	0.33	0.97 (0.95, 1.00)	0.06	0.98 (0.95, 1.01)	0.18
	RT			0.95 (0.87, 1.04)	0.29	0.92 (0.84, 1.00)	0.06	0.93 (0.84, 1.03)	0.18
IGP25	Continuous	32.83 (6.17)	32.58 (6.34)	1.01 (0.99, 1.02)	0.38	1.00 (0.99, 1.02)	0.67	1.01 (0.99, 1.02)	0.48
	RT			1.04 (0.95, 1.14)	0.37	1.02 (0.93, 1.12)	0.65	1.04 (0.94, 1.15)	0.47
IGP26	Continuous	16.35 (3.63)	17.35 (3.61)	0.94 (0.91, 0.96)	$7.3x10^{-7}$	0.93 (0.91, 0.96)	$2.7x10^{-7}$	0.94 (0.92, 0.97)	0.0003
	RT			0.79 (0.72, 0.86)	$1.6x10^{-7}$	0.77 (0.71, 0.85)	$6.2x10^{-8}$	0.81 (0.72, 0.90)	0.0001

IGP27	Continuous	21.21 (4.88)	21.82 (4.96)	0.98 (0.96, 1.00)	0.04	0.98 (0.96, 1.00)	0.03	0.99 (0.97, 1.01)	0.30
	RT			0.91 (0.83, 0.99)	0.04	0.91 (0.83, 0.99)	0.03	0.95 (0.86, 1.05)	0.30
IGP29	Continuous	40.18 (2.99)	39.51 (2.77)	1.07 (1.04, 1.10)	$1.2x10^{-5}$	1.03 (1.00, 1.06)	0.03	1.01 (0.98, 1.05)	0.47
	RT			1.23 (1.12, 1.35)	$9.2x10^{-6}$	1.12 (1.03, 1.23)	0.10	1.06 (0.95, 1.18)	0.28
IGP31	Continuous	37.02 (3.87)	36.41 (3.97)	1.04 (1.01, 1.06)	0.002	1.03 (1.01, 1.05)	0.006	1.03 (1.00, 1.05)	0.04
	RT			1.15 (1.05, 1.26)	0.002	1.14 (1.04, 1.24)	0.004	1.12 (1.01, 1.24)	0.03
**Bisecting GlcNAc**									
IGP36	Continuous	0.30 (0.08)	0.28 (0.07)	8.74 (2.87, 26.66)	0.0001	7.95 (2.43, 26.01)	0.001	9.40 (2.30, 38.49)	0.002
	RT			1.19 (1.09, 1.30)	0.0001	1.18 (1.07, 1.29)	0.001	1.19 (1.06, 1.33)	0.002
IGP37	Continuous	0.17 (0.05)	0.16 (0.05)	19.00 (3.31, 109.06)	0.001	19.22 (3.12, 118.60)	0.001	30.56 (3.50, 267.02)	0.002
	RT			1.16 (1.06, 1.27)	0.001	1.16 (1.06, 1.27)	0.002	1.18 (1.06, 1.32)	0.003
IGP38	Continuous	0.14 (0.04)	0.14 (0.03)	76.90 (6.38, 927.03)	0.001	73.95 (5.58, 980.16)	0.001	126.7 (5.94, 2701)	0.002
	RT			1.17 (1.07, 1.28)	0.001	1.16 (1.06, 1.28)	0.001	1.19 (1.06, 1.32)	0.002
IGP39	Continuous	1.35 (0.32)	1.27 (0.30)	1.82 (1.39, 2.39)	$1.5x10^{-5}$	1.66 (1.26, 2.19)	0.0003	1.62 (1.16, 2.27)	0.005
	RT			1.22 (1.11, 1.33)	$1.4x10^{-5}$	1.19 (1.09, 1.30)	0.0002	1.17 (1.05, 1.31)	0.005
IGP40	Continuous	0.56 (0.06)	0.55 (0.06)	30.19 (6.24, 146.08)	$2.3x10^{-5}$	19.39 (3.90, 96.34)	0.0003	13.96 (2.03, 96.03)	0.007
	RT			1.22 (1.11, 1.33)	$1.9x10^{-5}$	1.19 (1.08, 1.30)	0.0002	1.17 (1.05, 1.30)	0.005
**Neutral IgG glycans**									
IGP43	Continuous	32.33 (8.02)	29.56 (7.02)	1.04 (1.03, 1.05)	$3.7x10^{-12}$	1.04 (1.03, 1.05)	$1.2x10^{-10}$	1.03 (1.02, 1.05)	$1.0x10^{-5}$
	RT			1.36 (1.24, 1.49)	$4.3x10^{-11}$	1.35 (1.22, 1.48)	$6.9x10^{-10}$	1.28 (1.14, 1.43)	$2.6x10^{-5}$
IGP45	Continuous	7.78 (2.07)	7.18 (1.76)	1.13 (1.08, 1.18)	$4.9x10^{-8}$	1.12 (1.07, 1.17)	$2.0x10^{-6}$	1.09 (1.03, 1.15)	0.002
	RT			1.27 (1.16, 1.39)	$2.9x10^{-7}$	1.24 (1.13, 1.37)	$9.2x10^{-6}$	1.17 (1.05, 1.31)	0.006
IGP47	Continuous	22.59 (3.09)	23.32 (2.52)	0.92 (0.89, 0.95)	$1.1x10^{-6}$	0.93 (0.90, 0.96)	$5.0x10^{-6}$	0.93 (0.90, 0.97)	0.0002
	RT			0.81 (0.74, 0.88)	$4.7x10^{-6}$	0.81 (0.74, 0.89)	$1.4x10^{-5}$	0.83 (0.74, 0.92)	0.001
IGP48	Continuous	11.60 (1.81)	12.28 (1.75)	0.85 (0.81, 0.89)	$1.6x10^{-10}$	0.86 (0.82, 0.91)	$8.8x10^{-9}$	0.88 (0.83, 0.93)	$1.3x10^{-5}$
	RT			0.74 (0.68, 0.81)	$1.5x10^{-10}$	0.76 (0.70, 0.84)	$1.0x10^{-8}$	0.79 (0.71, 0.88)	$1.5x10^{-5}$
IGP49	Continuous	6.73 (1.54)	6.85 (1.46)	0.95 (0.89, 1.01)	0.08	0.94 (0.88, 1.00)	0.04	0.94 (0.87, 1.01)	0.09
	RT			0.91 (0.83, 1.00)	0.05	0.90 (0.82, 0.98)	0.02	0.90 (0.81, 1.01)	0.06
IGP53	Continuous	12.70 (4.29)	14.39 (4.37)	0.92 (0.90, 0.94)	$3.9x10^{-12}$	0.93 (0.91, 0.95)	$1.9x10^{-10}$	0.94 (0.92, 0.97)	$4.7x10^{-5}$
	RT			0.70 (0.64, 0.77)	$8.5x10^{-14}$	0.71 (0.65, 0.79)	$1.0x10^{-11}$	0.76 (0.68, 0.86)	$7.4x10^{-6}$
IGP54	Continuous	1.72 (0.54)	1.89 (0.59)	0.64 (0.54, 0.75)	$1.7x10^{-7}$	0.66 (0.55, 0.78)	$1.5x10^{-6}$	0.75 (0.61, 0.92)	0.005
	RT			0.77 (0.71, 0.85)	$3.4x10^{-8}$	0.79 (0.72, 0.87)	$4.5x10^{-7}$	0.85 (0.76, 0.95)	0.003

**Galactosylation**									
IGP55	Continuous	41.15 (9.19)	37.69 (8.09)	1.04 (1.03, 1.05)	$1.2x10^{-13}$	1.04 (1.03, 1.05)	$5.9x10^{-12}$	1.03 (1.02, 1.04)	$2.0x10^{-6}$
	RT			1.39 (1.27, 1.53)	$1.0x10^{-12}$	1.38 (1.26, 1.52)	$2.7x10^{-11}$	1.31 (1.16, 1.47)	$5.5x10^{-6}$
IGP56	Continuous	42.72 (4.71)	44.26 (3.56)	0.93 (0.91, 0.95)	$1.7x10^{-12}$	0.93 (0.92, 0.95)	$2.2x10^{-11}$	0.94 (0.92, 0.96)	$2.1x10^{-7}$
	RT			0.75 (0.69, 0.83)	$2.2x10^{-9}$	0.76 (0.69, 0.83)	$8.3x10^{-9}$	0.78 (0.69, 0.87)	$1.3x10^{-5}$
IGP57	Continuous	15.65 (5.00)	17.61 (5.15)	0.94 (0.92, 0.95)	$5.9x10^{-12}$	0.94 (0.92, 0.96)	$4.2x10^{-10}$	0.95 (0.93, 0.98)	0.0001
	RT			0.71 (0.65, 0.78)	$2.5x10^{-13}$	0.72 (0.66, 0.80)	$3.6x10^{-11}$	0.78 (0.69, 0.87)	$2.4x10^{-5}$
**Core fucosylation and bisecting GlcNAc**									
IGP62	Continuous	79.47 (3.74)	79.77 (3.46)	0.99 (0.96, 1.01)	0.27	0.99 (0.96, 1.02)	0.42	0.99 (0.96, 1.02)	0.41
	RT			0.96 (0.87, 1.05)	0.32	0.97 (0.88, 1.06)	0.52	0.96 (0.86, 1.07)	0.48
IGP63	Continuous	78.81 (4.29)	78.69 (4.11)	1.01 (0.99, 1.03)	0.34	1.01 (0.99, 1.03)	0.29	1.01 (0.98, 1.04)	0.51
	RT			1.05 (0.96, 1.15)	0.30	1.06 (0.96, 1.16)	0.24	1.04 (0.93, 1.16)	0.46
IGP64	Continuous	80.03 (3.79)	80.45 (3.56)	0.98 (0.96, 1.00)	0.10	0.98 (0.96, 1.01)	0.18	0.98 (0.95, 1.01)	0.25
	RT			0.93 (0.85, 1.02)	0.12	0.95 (0.86, 1.04)	0.24	0.94 (0.85, 1.05)	0.31
IGP66	Continuous	17.29 (3.20)	16.97 (2.90)	1.02 (0.99, 1.05)	0.14	1.01 (0.98, 1.05)	0.38	1.01 (0.97, 1.05)	0.64
	RT			1.06 (0.97, 1.16)	0.19	1.03 (0.94, 1.13)	0.50	1.02 (0.91, 1.14)	0.73
IGP67	Continuous	19.15 (3.88)	19.30 (3.65)	0.99 (0.96, 1.01)	0.26	0.98 (0.96, 1.01)	0.17	0.98 (0.96, 1.01)	0.28
	RT			0.95 (0.86, 1.03)	0.22	0.93 (0.85, 1.02)	0.13	0.94 (0.84, 1.05)	0.25
IGP68	Continuous	18.20 (3.46)	17.82 (3.25)	1.02 (0.99, 1.05)	0.12	1.02 (0.99, 1.04)	0.27	1.01 (0.98, 1.05)	0.44
	RT			1.07 (0.98, 1.17)	0.13	1.05 (0.95, 1.15)	0.34	1.04 (0.93, 1.16)	0.50
IGP70	Continuous	0.22 (0.05)	0.21 (0.05)	4.33 (0.71, 26.41)	0.11	2.73 (0.41, 18.15)	0.30	2.05 (0.23, 18.63)	0.52
	RT			1.06 (0.97, 1.16)	0.19	1.03 (0.94, 1.14)	0.49	1.02 (0.92, 1.14)	0.68
IGP71	Continuous	17.88 (3.38)	17.55 (3.07)	1.02 (0.99, 1.05)	0.16	1.01 (0.98, 1.04)	0.38	1.01 (0.98, 1.04)	0.59
	RT			1.06 (0.97, 1.16)	0.21	1.03 (0.94, 1.14)	0.49	1.02 (0.92 , 1.14)	0.68
IGP72	Continuous	4.65 (1.09)	4.71 (1.01)	0.97 (0.89, 1.06)	0.51	1.00 (0.91, 1.09)	0.93	0.99 (0.89, 1.10)	0.85
	RT			0.96 (0.87, 1.05)	0.32	0.98 (0.89, 1.07)	0.64	0.98 (0.88, 1.09)	0.74

Supplementary Table 3 CRC-specific analysis

Code	Glycan	Dead (N=385)	Survived (N=844)	Crude model (n=1229)		Model I (AJCC, age, sex, n=1229)		Model II (AJCC, age, sex, time between sample and surgery, operation type, CRP, bmi, n=971)	
		Mean (SD)	Mean (SD)	HR (95% CI)	p-value	HR (95% CI)	p-value	HR (95% CI)	p-value
**Total IgG glycans (neutral and charged); Measured**									
IGP3	Continuous	26.20 (7.31)	24.27 (6.59)	1.04 (1.02, 1.05)	$5.9x10^{-8}$	1.04 (1.03, 1.06)	$2.2x10^{-8}$	1.03 (1.01, 1.05)	0.001
	RT			1.31 (1.18, 1.45)	$2.8x10^{-7}$	1.33 (1.20, 1.48)	$5.5x10^{-8}$	1.23 (1.09, 1.40)	0.001
IGP5	Continuous	6.23 (1.85)	5.91 (1.63)	1.10 (1.04, 1.17)	0.0004	1.12 (1.05, 1.18)	0.0002	1.06 (0.99, 1.14)	0.10
	RT			1.18 (1.07, 1.31)	0.001	1.22 (1.09, 1.35)	0.0003	1.10 (0.97, 1.25)	0.14
IGP7	Continuous	18.33 (2.13)	18.59 (1.91)	0.94 (0.89, 0.99)	0.01	0.94 (0.89, 0.99)	0.01	0.95 (0.89, 1.01)	0.10
	RT			0.89 (0.80, 0.99)	0.03	0.89 (0.80, 0.98)	0.02	0.92 (0.81, 1.04)	0.16
IGP8	Continuous	9.46 (1.35)	9.74 (1.38)	0.87 (0.81, 0.94)	0.003	0.90 (0.84, 0.97)	0.006	0.92 (0.84, 1.00)	0.05
	RT			0.83 (0.75, 0.92)	0.0003	0.88 (0.79, 0.97)	0.009	0.90 (0.80, 1.01)	0.06
IGP9	Continuous	5.40 (1.19)	5.50 (1.18)	0.93 (0.85, 1.01)	0.10	0.93 (0.85, 1.02)	0.11	0.92 (0.83, 1.03)	0.15
	RT			0.91 (0.82, 1.01)	0.08	0.91 (0.82, 1.01)	0.07	0.90 (0.80, 1.02)	0.11
IGP13	Continuous	10.30 (3.19)	11.24 (3.15)	0.91 (0.88, 0.94)	$3.5x10^{-8}$	0.91 (0.88, 0.94)	$5.3x10^{-8}$	0.94 (0.90, 0.98)	0.004
	RT			0.73 (0.66, 0.81)	$5.3x10^{-9}$	0.73 (0.66, 0.81)	$1.5x10^{-8}$	0.81 (0.71, 0.92)	0.002
IGP14	Continuous	1.38 (0.40)	1.49 (0.43)	0.54 (0.42, 0.70)	$2.9x10^{-6}$	0.56 (0.43, 0.72)	$1.2x10^{-5}$	0.70 (0.51, 0.96)	0.03
	RT			0.77 (0.70, 0.86)	$9.7x10^{-7}$	0.79 (0.71, 0.87)	$5.7x10^{-6}$	0.86 (0.76, 0.98)	0.02
IGP17	Continuous	7.87 (2.31)	8.35 (2.34)	0.91 (0.87, 0.96)	0.0001	0.90 (0.85, 0.94)	$5.2x10^{-6}$	0.92 (0.87, 0.97)	0.003
	RT			0.80 (0.72, 0.89)	$2.4x10^{-5}$	0.77 (0.70, 0.86)	$1.4x10^{-6}$	0.82 (0.72, 0.93)	0.002
IGP18	Continuous	1.87 (0.37)	1.90 (0.39)	0.85 (0.65, 1.10)	0.22	0.78 (0.60, 1.02)	0.07	0.99 (0.74, 1.33)	0.97
	RT			0.94 (0.85, 1.04)	0.24	0.92 (0.83, 1.01)	0.08	1.00 (0.90, 1.12)	0.98
**Sialylation; Derived**									
IGP24	Continuous	24.84 (3.24)	24.90 (3.09)	0.99 (0.96, 1.02)	0.63	0.97 (0.94, 1.00)	0.06	0.97 (0.93, 1.00)	0.09
	RT			0.97 (0.88, 1.07)	0.56	0.91 (0.83, 1.01)	0.07	0.90 (0.80, 1.01)	0.08
IGP25	Continuous	32.93 (6.13)	32.56 (6.34)	1.01 (0.99, 1.03)	0.27	1.00 (0.99, 1.02)	0.67	1.01 (0.99, 1.03)	0.37
	RT			1.06 (0.96, 1.17)	0.26	1.02 (0.92, 1.13)	0.65	1.05 (0.94, 1.19)	0.38
IGP26	Continuous	16.47 (3.64)	17.17 (3.63)	0.95 (0.92, 0.98)	0.0003	0.94 (0.91, 0.96)	$7.4x10^{-6}$	0.95 (0.92, 0.98)	0.002
	RT			0.82 (0.74, 0.91)	0.0001	0.78 (0.71, 0.87)	$3.4x10^{-6}$	0.82 (0.72, 0.92)	0.001

IGP27	Continuous	21.38 (4.93)	21.66 (4.94)	0.99 (0.97, 1.01)	0.29	0.98 (0.96, 1.00)	0.08	1.00 (0.97, 1.02)	0.79
	RT			0.95 (0.86, 1.05)	0.30	0.92 (0.83, 1.01)	0.09	0.99 (0.88, 1.11)	0.80
IGP29	Continuous	40.22 (3.05)	39.57 (2.77)	1.07 (1.04, 1.11)	$4.0x10^{-5}$	1.02 (0.99, 1.06)	0.13	0.99 (0.96, 1.03)	0.75
	RT			1.24 (1.12, 1.38)	$3.7x10^{-5}$	1.10 (1.00, 1.22)	0.05	1.00 (0.89., 1.13)	0.99
IGP31	Continuous	37.11 (3.80)	36.44 (3.99)	1.04 (1.01, 1.07)	0.002	1.04 (1.01, 1.06)	0.006	1.04 (1.01, 1.07)	0.01
	RT			1.18 (1.06, 1.30)	0.001	1.15 (1.05, 1.27)	0.004	1.17 (1.04, 1.31)	0.009
**Bisecting GlcNAc; Derived**									
IGP36	Continuous	0.30 (0.07)	0.29 (0.08)	4.42 (1.23, 15.89)	0.02	5.06 (1.30, 19.60)	0.02	7.14 (1.41, 36.18)	0.02
	RT			1.13 (1.03, 1.25)	0.01	1.14 (1.03, 1.27)	0.01	1.17 (1.03, 1.33)	0.02
IGP37	Continuous	0.17 (0.05)	0.16 (0.05)	7.92 (1.06, 58.84)	0.04	11.26 (1.42, 89.56)	0.02	30.51 (2.53, 367.34)	0.007
	RT			1.12 (1.01, 1.23)	0.03	1.13 (1.02, 1.26)	0.02	1.19 (1.05, 1.35)	0.006
IGP38	Continuous	0.14 (0.03)	0.14 (0.03)	24.79 (1.45, 423.15)	0.03	40.48 (2.19, 748.37)	0.01	138.34 (4.24, 4509)	0.006
	RT			1.12 (1.02, 1.24)	0.02	1.14 (1.03, 1.27)	0.01	1.19 (1.06, 1.35)	0.005
IGP39	Continuous	1.33 (0.32)	1.29 (0.30)	1.53 (1.12, 2.08)	0.008	1.42 (1.04, 1.94)	0.03	1.35 (0.92, 1.98)	0.13
	RT			1.15 (1.04, 1.27)	0.006	1.13 (1.02, 1.25)	0.02	1.10 (0.98, 1.25)	0.12
IGP40	Continuous	0.56 (0.06)	0.55 (0.06)	10.50 (1.78, 61.78)	0.009	7.90 (1.32, 47.39)	0.02	4.87 (0.55, 43.15)	0.16
	RT			1.15 (1.04, 1.27)	0.008	1.13 (1.02, 1.25)	0.02	1.10 (0.97, 1.25)	0.13
**Neutral IgG glycans; Derived**									
IGP43	Continuous	32.14 (8.00)	29.98 (7.24)	1.04 (1.02, 1.05)	$3.5x10^{-8}$	1.02 (1.03, 1.05)	$2.8x10^{-8}$	1.03 (1.01, 1.05)	0.001
	RT			1.32 (1.19, 1.46)	$1.6x10^{-7}$	1.33 (1.20, 1.48)	$6.4x10^{-8}$	1.24 (1.09, 1.41)	0.001
IGP45	Continuous	7.65 (2.07)	7.31 (1.83)	1.09 (1.04, 1.14)	0.0009	1.10 (1.04, 1.15)	0.001	1.04 (0.98, 1.11)	0.19
	RT			1.17 (1.06, 1.30)	0.002	1.19 (1.07, 1.33)	0.001	1.08 (0.94, 1.22)	0.27
IGP47	Continuous	22.71 (3.01)	23.18 (2.66)	0.94 (0.91, 0.98)	0.001	0.93 (0.90, 0.97)	0.0002	0.95 (0.91, 0.99)	0.02
	RT			0.85 (0.77, 0.95)	0.003	0.83 (0.75, 0.92)	0.0003	0.87 (0.77, 0.98)	0.02
IGP48	Continuous	11.72 (1.81)	12.14 (1.79)	0.89 (0.84, 0.94)	$2.2x10^{-5}$	0.90 (0.85, 0.95)	0.0002	0.92 (0.86, 0.98)	0.01
	RT			0.80 (0.72, 0.89)	$2.1x10^{-5}$	0.82 (0.74, 0.91)	0.0002	0.85 (0.76, 0.97)	0.01
IGP49	Continuous	6.68 (1.49)	6.85 (1.50)	0.92 (0.86, 0.99)	0.03	0.91 (0.85, 0.98)	0.01	0.92 (0.84, 1.00)	0.04
	RT			0.89 (0.80, 0.98)	0.02	0.87 (0.78, 0.97)	0.009	0.88 (0.77, 0.99)	0.03
IGP53	Continuous	12.86 (4.38)	14.11 (4.38)	0.93 (0.91, 0.96)	$1.1x10^{-7}$	0.93 (0.91, 0.96)	$5.6x10^{-8}$	0.95 (0.93, 0.98)	0.003
	RT			0.74 (0.67, 0.82)	$9.1x10^{-9}$	0.73 (0.66, 0.81)	$9.7x10^{-9}$	0.80 (0.71, 0.92)	0.001
IGP54	Continuous	1.72 (0.53)	1.87 (0.59)	0.63 (0.52, 0.76)	$2.4x10^{-6}$	0.63 (0.52, 0.77)	$3.9x10^{-6}$	0.76 (0.60, 0.95)	0.02

	RT			0.77 (0.70, 0.86)	8.5x10^{-11}	0.78 (0.70, 0.86)	2.0x10^{-6}	0.85 (0.75, 0.97)	0.01
**Galactosylation; Derived**									
IGP55	Continuous	40.83 (9.18)	38.26 (8.36)	1.03 (1.02, 1.04)	1.7x10^{-8}	1.03 (1.02, 1.05)	1.0x10^{-8}	1.02 (1.01, 1.04)	0.001
	RT			1.33 (1.20, 1.47)	7.0x10^{-8}	1.35 (1.22, 1.50)	2.3x10^{-8}	1.24 (1.09, 1.41)	0.001
IGP56	Continuous	42.90 (4.63)	43.99 (3.83)	0.94 (0.92, 0.96)	2.5x10^{-7}	0.94 (0.92, 0.96)	1.1x10^{-7}	0.95 (0.92, 0.98)	0.0004
	RT			0.80 (0.72, 0.88)	1.5x10^{-5}	0.78 (0.70, 0.86)	2.4x10^{-6}	0.82 (0.72, 0.94)	0.003
IGP57	Continuous	15.79 (5.09)	17.30 (5.16)	0.94 (0.92, 0.96)	6.5x10^{-8}	0.94 (0.92, 0.96)	6.1x10^{-8}	0.96 (0.94, 0.99)	0.005
	RT			0.74 (0.66, 0.82)	7.0x10^{-9}	0.73 (0.66, 0.82)	1.2x10^{-8}	0.81 (0.71, 0.93)	0.002
**Core fucosylation and bisecting GlcNAc; Derived**									
IGP62	Continuous	79.68 (3.70)	79.63 (3.52)	1.00 (0.98, 1.03)	0.79	1.00 (0.97, 1.03)	0.94	1.00 (0.97, 1.04)	0.88
	RT			1.02 (0.92, 1.13)	0.71	1.01 (0.91, 1.13)	0.80	1.02 (0.90, 1.15)	0.77
IGP63	Continuous	79.01 (4.23)	78.62 (4.16)	1.02 (1.00, 1.05)	0.07	1.02 (1.00, 1.05)	0.10	1.02 (0.99, 1.05)	0.17
	RT			1.10 (1.00, 1.22)	0.06	1.10 (0.99, 1.22)	0.07	1.10 (0.97, 1.24)	0.13
IGP64	Continuous	80.24 (3.73)	80.30 (3.62)	1.00 (0.97, 1.02)	0.84	1.00 (0.97, 1.02)	0.75	1.00 (0.96, 1.03)	0.94
	RT			0.99 (0.90, 1.10)	0.92	0.99 (0.89, 1.11)	0.90	1.00 (0.89, 1.14)	0.94
IGP66	Continuous	17.10 (3.15)	17.09 (2.97)	1.00 (0.97, 1.03)	0.99	1.00 (0.96, 1.03)	0.95	0.99 (0.95, 1.03)	0.51
	RT			0.99 (0.90, 1.10)	0.89	0.99 (0.89, 1.10)	0.80	0.95 (0.84, 1.08)	0.44
IGP67	Continuous	18.98 (3.81)	19.36 (3.70)	0.97 (0.95, 1.00)	0.06	0.97 (0.95, 1.00)	0.06	0.97 (0.94, 1.00)	0.08
	RT			0.90 (0.82, 1.00)	0.05	0.90 (0.81, 1.00)	0.04	0.89 (0.79, 1.01)	0.07
IGP68	Continuous	18.01 (3.39)	17.96 (3.32)	1.00 (0.97, 1.03)	0.87	1.00 (0.97, 1.03)	0.93	0.99 (0.96, 1.03)	0.73
	RT			1.00 (0.91, 1.11)	0.94	1.00 (0.90, 1.11)	0.93	0.97 (0.86, 1.10)	0.64
IGP70	Continuous	0.21 (0.05)	0.21 (0.05)	1.08 (0.13, 8.62)	0.94	1.06 (0.12, 9.53)	0.30	0.50 (0.04, 6.76)	0.60
	RT			0.99 (0.90, 1.10)	0.87	0.99 (0.89, 1.10)	0.79	0.95 (0.84, 1.08)	0.47
IGP71	Continuous	17.68 (3.33)	17.68 (3.14)	1.00 (0.97, 1.03)	0.96	1.00 (0.97, 1.03)	0.96	0.99 (0.95, 1.03)	0.58
	RT			0.99 (0.89, 1.10)	0.85	0.99 (0.89, 1.10)	0.80	0.96 (0.85, 1.09)	0.50
IGP72	Continuous	4.71 (1.06)	4.68 (1.03)	1.03 (0.93, 1.13)	0.61	1.03 (0.93, 1.14)	0.54	1.05 (0.93, 1.18)	0.42
	RT			1.02 (0.92, 1.13)	0.68	1.02 (0.92, 1.14)	0.67	1.05 (0.92, 1.18)	0.47

Supplementary Table 4 All-cause mortality analysis for stages 1-3

Code	Glycan	Dead (N=355)	Survived (N=728)	Crude model (n=1083)		Model I (AJCC, age, sex, n=1083)		Model II (AJCC, age, sex, time between sample and surgery, operation type, bmi, CRP n=850)	
		**Mean (SD)**	**Mean (SD)**	**HR (95% CI)**	**p-value**	**HR (95% CI)**	**p-value**	**HR (95% CI)**	**p-value**
**Total IgG glycans (neutral and charged); Measured**									
IGP3	Continuous	26.11 (7.30)	23.90 (6.34)	1.04 (1.03, 1.06)	$9.5x10^{-8}$	1.04 (1.02, 1.05)	$1.6x10^{-6}$	1.04 (1.02, 1.06)	$9.6x10^{-5}$
	RT			1.32 (1.18, 1.47)	$4.4x10^{-7}$	1.29 (1.16, 1.45)	$8.13x10^{-6}$	1.28 (1.11, 1.46)	0.0004
IGP5	Continuous	6.43 (1.86)	5.80 (1.86)	1.18 (1.12, 1.25)	$3.3x10^{-9}$	1.16 (1.10, 1.24)	$7.6x10^{-7}$	1.16 (1.08, 1.25)	$3.8x10^{-5}$
	RT			1.35 (1.22, 1.51)	$3.0x10^{-8}$	1.30 (1.16, 1.47)	$7.2x10^{-6}$	1.30 (1.13, 1.49)	0.0002
IGP7	Continuous	18.25 (2.18)	18.66 (1.80)	0.91 (0.86, 0.96)	0.001	0.92 (0.87, 0.98)	0.006	0.91 (0.85, 0.97)	0.002
	RT			0.84 (0.75, 0.93)	0.001	0.87 (0.78, 0.97)	0.01	0.84 (0.74, 0.96)	0.008
IGP8	Continuous	9.35 (1.39)	9.84 (1.34)	0.81 (0.75, 0.87)	$5.9x10^{-8}$	0.81 (0.75, 0.88)	$2.1x10^{-7}$	0.80 (0.73, 0.87)	$7.9x10^{-7}$
	RT			0.75 (0.67, 0.83)	$6.9x10^{-8}$	0.75 (0.68, 0.84)	$2.8x10^{-7}$	0.73 (0.65, 0.83)	$1.0x10^{-6}$
IGP9	Continuous	5.57 (1.27)	5.48 (1.16)	1.05 (0.96, 1.14)	0.32	1.02 (0.93, 1.12)	0.65	1.00 (0.91, 1.11)	0.94
	RT			1.04 (0.94, 1.16)	0.44	1.01 (0.91, 1.13)	0.81	1.00 (0.88, 1.13)	0.95
IGP13	Continuous	10.29 (3.07)	11.42 (3.11)	0.90 (0.87, 0.93)	$8.1x10^{-9}$	0.91 (0.87, 0.94)	$7.9x10^{-7}$	0.92 (0.88, 0.96)	0.0003
	RT			0.71 (0.64, 0.80)	$1.6x10^{-9}$	0.73 (0.65, 0.82)	$1.6x10^{-7}$	0.75 (0.65, 0.87)	$7.5x10^{-5}$
IGP14	Continuous	1.41 (0.41)	1.50 (0.43)	0.64 (0.49, 0.83)	0.001	0.67 (0.51, 0.87)	0.003	0.73 (0.53, 1.00)	0.05
	RT			0.83 (0.74, 0.92)	0.0004	0.84 (0.75, 0.94)	0.002	0.87 (0.76, 0.99)	0.03
IGP17	Continuous	7.83 (2.30)	8.45 (2.28)	0.90 (0.86, 0.94)	$2.6x10^{-5}$	0.91 (0.86, 0.96)	0.0005	0.92 (0.87, 0.99)	0.02
	RT			0.77 (0.70, 0.86)	$3.2x10^{-6}$	0.79 (0.70, 0.89)	$5.7x10^{-5}$	0.81 (0.71, 0.93)	0.003
IGP18	Continuous	1.88 (0.39)	1.90 (0.39)	0.89 (0.67, 1.16)	0.38	0.87 (0.67, 1.13)	0.31	0.89 (0.66, 1.20)	0.43
	RT			0.95 (0.86, 1.05)	0.33	0.95 (0.86, 1.05)	0.33	0.95 (0.85, 1.07)	0.40
**Sialylation**									
IGP24	Continuous	24.78 (3.18)	24.93 (3.04)	0.99 (0.95, 1.02)	0.49	0.99 (0.96, 1.03)	0.58	1.00 (0.96, 1.04)	0.88
	RT			0.96 (0.86, 1.06)	0.40	0.96 (0.86, 1.07)	0.48	1.00 (0.88, 1.13)	0.99
IGP25	Continuous	32.49 (6.30)	32.58 (6.38)	1.00 (0.98, 1.02)	0.91	1.00 (0.98, 1.02)	0.94	1.00 (0.98, 1.02	0.90
	RT			0.99 (0.90, 1.10)	0.91	1.00 (0.90, 1.10)	0.96	1.01 (0.90, 1.13)	0.89
IGP26	Continuous	16.43 (3.64)	17.32 (3.55)	0.94 (0.91, 0.97)	0.0001	0.95 (0.92, 0.98)	0.001	0.95 (0.92, 0.99)	0.02
	RT			0.80 (0.72, 0.89)	$4.0x10^{-5}$	0.81 (0.73, 0.91)	0.0002	0.83 (0.73, 0.95)	0.007

IGP27	Continuous	21.01 (5.01)	21.81 (4.97)	0.97 (0.95, 1.00)	0.02	0.98 (0.96, 1.00)	0.05	0.98 (0.96, 1.01)	0.14
	RT			0.87 (0.79, 0.97)	0.01	0.90 (0.81, 1.00)	0.05	0.91 (0.81, 1.02)	0.12
IGP29	Continuous	40.09 (2.90)	39.49 (2.70)	1.07 (1.03, 1.11)	0.0001	1.06 (1.02, 1.10)	0.001	1.06 (1.01, 1.10)	0.009
	RT			1.23 (1.10, 1.37)	0.0002	1.20 (1.07, 1.34)	0.001	1.19 (1.05, 1.35)	0.007
IGP31	Continuous	36.78 (3.86)	36.40 (3.97)	1.02 (1.00, 1.05)	0.10	1.02 (0.99, 1.05)	0.12	1.01 (0.98, 1.04)	0.58
	RT			1.10 (0.99, 1.21)	0.08	1.09 (0.98, 1.21)	0.10	1.04 (0.92, 1.17)	0.53
**Bisecting GlcNAc**									
IGP36	Continuous	0.30 (0.08)	0.28 (0.07)	10.21 (2.80, 37.30)	0.0004	7.18 (1.82, 28.32)	0.005	7.26 (1.42, 37.22)	0.02
	RT			1.20 (1.09, 1.34)	0.0004	1.17 (1.05, 1.30)	0.006	1.16 (1.02, 1.32)	0.02
IGP37	Continuous	0.17 (0.05)	0.16 (0.05)	20.26 (2.63, 155.9)	0.004	13.32 (1.57, 112.9)	0.02	11.89 (0.94, 150.5)	0.06
	RT			1.16 (1.05, 1.29)	0.005	1.13 (1.02, 1.26)	0.02	1.12 (0.99, 1.28)	0.08
IGP38	Continuous	0.14 (0.04)	0.14 (0.03)	84.20 (4.58, 1549)	0.003	43.03 (2.04, 909.9)	0.02	33.95 (0.92, 12.46)	0.06
	RT			1.17 (1.05, 1.30)	0.003	1.14 (1.02, 1.27)	0.02	1.13 (0.99, 1.28)	0.07
IGP39	Continuous	1.36 (0.33)	1.27 (0.30)	2.04 (1.49, 2.81)	$1.0x10^{-5}$	1.86 (1.34, 2.58)	0.0002	1.84 (1.24, 2.73)	0.002
	RT			1.26 (1.13, 1.40)	$2.0x10^{-5}$	1.22 (1.10, 1.36)	0.0003	1.21 (1.06, 1.37)	0.005
IGP40	Continuous	0.56 (0.06)	0.55 (0.06)	55.05 (8.57, 353.6)	$2.4x10^{-5}$	32.58 (4.82, 220.4)	0.0004	24.66 (2.52, 241.26)	0.006
	RT			1.26 (1.13, 1.40)	$2.2x10^{-5}$	1.22 (1.09, 1.36)	0.0003	1.20 (1.06, 1.37)	0.005
**Neutral IgG glycans**									
IGP43	Continuous	31.97 (7.93)	29.59 (6.98)	1.04 (1.02, 1.05)	$1.3x10^{-7}$	1.04 (1.02, 1.05)	$2.4x10^{-6}$	1.03 (1.02, 1.05)	0.0001
	RT			1.32 (1.18, 1.47)	$5.3x10^{-7}$	1.29 (1.15, 1.45)	$1.0x10^{-5}$	1.28 (1.12, 1.47)	0.0004
IGP45	Continuous	7.88 (2.08)	7.18 (1.75)	1.16 (1.10, 1.22)	$5.4x10^{-9}$	1.14 (1.08, 1.21)	$1.6x10^{-6}$	1.14 (1.07, 1.22)	$5.8x10^{-5}$
	RT			1.35 (1.21, 1.50)	$4.8x10^{-8}$	1.29 (1.15, 1.45)	$1.5x10^{-5}$	1.29 (1.12, 1.48)	0.0003
IGP47	Continuous	22.58 (3.07)	23.30 (2.50)	0.92 (0.88, 0.95)	$1.3x10^{-5}$	0.93 (0.89, 0.97)	0.0004	0.92 (0.88, 0.97)	0.0004
	RT			0.79 (0.71, 0.89)	$3.8x10^{-5}$	0.82 (0.73, 0.92)	0.001	0.81 (0.71, 0.92)	0.002
IGP48	Continuous	11.56 (1.84)	12.29 (1.74)	0.83 (0.78, 0.88)	$8.8x10^{-10}$	0.84 (0.79, 0.89)	$6.5x10^{-9}$	0.83 (0.77, 0.89)	$1.1x10^{-7}$
	RT			0.72 (0.64, 0.80)	$8.0x10^{-10}$	0.72 (0.65, 0.81)	$7.0x10^{-9}$	0.71 (0.63, 0.81)	$1.2x10^{-7}$
IGP49	Continuous	6.88 (1.60)	6.84 (1.47)	1.01 (0.94, 1.08)	0.80	0.99 (0.93, 1.07)	0.88	0.99 (0.91, 1.07)	0.73
	RT			1.00 (0.90, 1.12)	0.95	0.98 (0.88, 1.09)	0.72	0.97 (0.86, 1.10)	0.63
IGP53	Continuous	12.82 (4.23)	14.36 (4.34)	0.93 (0.90, 0.95)	$2.0x10^{-8}$	0.93 (0.91, 0.96)	$1.5x10^{-6}$	0.94 (0.91, 0.97)	0.001
	RT			0.72 (0.64, 0.80)	$1.8x10^{-9}$	0.73 (0.65, 0.82)	$1.5x10^{-7}$	0.75 (0.65, 0.87)	$7.8x10^{-5}$
IGP54	Continuous	1.76 (0.55)	1.88 (0.59)	0.70 (0.58, 0.85)	0.0004	0.73 (0.60, 0.89)	0.002	0.78 (0.62, 0.99)	0.04
	RT			0.82 (0.73, 0.91)	0.0002	0.83 (0.75, 0.93)	0.001	0.86 (0.76, 0.98)	0.02

**Galactosylation**									
IGP55	Continuous	40.89 (9.04)	37.72 (8.04)	1.04 (1.03, 1.05)	1.7x10^{-9}	1.04 (1.02, 1.05)	7.1x10^{-8}	1.04 (1.02, 1.05)	8.4x10^{-6}
	RT			1.38 (1.24, 1.54)	6.7x10^{-9}	1.35 (1.21, 1.52)	2.9x10^{-7}	1.34 (1.17, 1.54)	2.9x10^{-5}
IGP56	Continuous	42.83 (4.68)	42.25 (3.54)	0.93 (0.91, 0.96)	6.5x10^{-9}	0.94 (0.91, 0.96)	6.9x10^{-8}	0.93 (0.90, 0.96)	3.3x10^{-7}
	RT			0.76 (0.68, 0.85)	9.3x10^{-7}	0.77 (0.69, 0.87)	1.0x10^{-5}	0.75 (0.65, 0.86)	2.6x10^{-5}
IGP57	Continuous	15.81 (4.93)	17.58 (5.12)	0.94 (0.92, 0.96)	4.1x10^{-8}	0.94 (0.92, 0.97)	2.5x10^{-6}	0.95 (0.93, 0.98)	0.001
	RT			0.72 (0.65, 0.81)	5.4x10^{-9}	0.74 (0.66, 0.83)	3.6x10^{-7}	0.76 (0.66, 0.88)	0.0002
**Core fucosylation and bisecting GlcNAc**									
IGP62	Continuous	79.17 (3.89)	79.77 (3.47)	0.97 (0.94, 0.99)	0.02	0.98 (0.95, 1.01)	0.11	0.97 (0.94, 1.01)	0.12
	RT			0.89 (0.80, 0.99)	0.03	0.92 (0.83, 1.03)	0.15	0.91 (0.81, 1.03)	0.15
IGP63	Continuous	78.42 (4.42)	78.69 (4.14)	0.99 (0.97, 1.01)	0.41	1.00 (0.97, 1.02)	0.74	0.99 (0.96, 1.02)	0.54
	RT			0.96 (0.86, 1.07)	0.46	0.99 (0.89, 1.10)	0.81	0.96 (0.85, 1.09)	0.57
IGP64	Continuous	79.71 (3.94)	80.45 (3.57)	0.96 (0.93, 0.99)	0.003	0.97 (0.94, 1.00)	0.03	0.97 (0.94, 1.00)	0.04
	RT			0.86 (0.77, 0.96)	0.005	0.89 (0.80, 0.99)	0.04	0.88 (0.78, 1.00)	0.05
IGP66	Continuous	17.58 (3.31)	16.97 (2.91)	1.05 (1.02, 1.09)	0.002	1.04 (1.00, 1.07)	0.04	1.04 (1.00, 1.08)	0.08
	RT			1.16 (1.05, 1.29)	0.005	1.11 (1.00, 1.24)	0.06	1.11 (0.98, 1.26)	0.10
IGP67	Continuous	19.52 (3.98)	19.29 (3.67)	1.01 (0.98, 1.04)	0.44	1.00 (0.98, 1.03)	0.83	1.01 (0.97, 1.04)	0.67
	RT			1.04 (0.93, 1.15)	0.51	1.00 (0.90, 1.12)	0.93	1.02 (0.90, 1.16)	0.71
IGP68	Continuous	18.52 (3.59)	17.82 (3.27)	1.05 (1.02, 1.08)	0.002	1.04 (1.01, 1.07)	0.02	1.04 (1.00, 1.08)	0.04
	RT			1.17 (1.05, 1.30)	0.003	1.13 (1.01, 1.25)	0.03	1.13 (1.00, 1.28)	0.05
IGP70	Continuous	0.22 (0.05)	0.21 (0.05)	27.42 (3.53, 213.1)	0.002	10.93 (1.32, 90.70)	0.03	10.14 (0.88, 116.8)	0.06
	RT			1.16 (1.05, 1.29)	0.005	1.11 (0.99, 1.24)	0.06	1.11 (0.98, 1.26)	0.10
IGP71	Continuous	18.18 (3.50)	17.55 (3.08)	1.05 (1.02, 1.08)	0.003	1.03 (1.00, 1.07)	0.04	1.03 (1.00, 1.07)	0.08
	RT			1.16 (1.04, 1.29)	0.006	1.10 (0.99, 1.23)	0.07	1.11 (0.98, 1.26)	0.11
IGP72	Continuous	4.57 (1.10)	4.72 (1.01)	0.90 (0.81, 1.00)	0.04	0.94 (0.84, 1.05)	0.25	0.92 (0.81, 1.04)	0.19
	RT			0.88 (0.79, 0.97)	0.01	0.92 (0.82, 1.02)	0.12	0.91 (0.80, 1.03)	0.15

Supplementary Table 5 CRC-specific mortality analysis for stages 1-3

Code	Glycan	Dead (N=257)	Survived (N=826)	Crude model (n=1083)		Model I (AJCC, age, sex, n=1083)		Model II (AJCC, age, sex, time between sample and surgery, operation type, bmi, CRP n=850)	
		Mean (SD)	Mean (SD)	HR (95% CI)	p-value	HR (95% CI)	p-value	HR (95% CI)	p-value
**Total IgG glycans (neutral and charged); Measured**									
IGP3	Continuous	25.76 (7.28)	24.27 (6.53)	1.03 (1.01, 1.05)	0.0004	1.04 (1.02, 1.05)	0.0001	1.03 (1.01, 1.06)	0.004
	RT			1.24 (1.10, 1.40)	0.001	1.28 (1.12, 1.46)	0.002	1.24 (1.05, 1.45)	0.01
IGP5	Continuous	6.29 (1.85)	5.91 (1.63)	1.13 (1.05, 1.21)	0.005	1.13 (1.05, 1.22)	0.001	1.10 (1.01, 1.20)	0.04
	RT			1.23 (1.09, 1.40)	0.001	1.23 (1.08, 1.42)	0.002	1.17 (0.99, 1.37)	0.06
IGP7	Continuous	18.33 (2.09)	18.59 (1.90)	0.93 (0.88, 1.00)	0.04	0.94 (0.88, 1.00)	0.05	0.93 (0.86, 1.00)	0.07
	RT			0.89 (0.78, 1.01)	0.07	0.89 (0.78, 1.02)	0.09	0.88 (0.76, 1.03)	0.11
IGP8	Continuous	9.46 (1.38)	9.75 (1.37)	0.86 (0.79, 0.94)	0.001	0.86 (0.79, 0.95)	0.002	0.84 (0.75, 0.93)	0.001
	RT			0.82 (0.72, 0.92)	0.001	0.82 (0.73, 0.93)	0.002	0.79 (0.68, 0.91)	0.001
IGP9	Continuous	5.54 (1.25)	5.50 (1.18)	1.02 (0.92, 1.13)	0.67	0.99 (0.89, 1.10)	0.83	0.97 (0.85, 1.10)	0.59
	RT			1.02 (0.90, 1.15)	0.77	0.98 (0.86, 1.11)	0.73	0.95 (0.82, 1.10)	0.52
IGP13	Continuous	10.48 (3.14)	11.23 (3.13)	0.92 (0.89, 0.96)	0.0001	0.91 (0.87, 0.96)	$8.3x10^{-5}$	0.93 (0.88, 0.99)	0.02
	RT			0.77 (0.68, 0.87)	$6.0x10^{-5}$	0.74 (0.65, 0.85)	$3.1x10^{-5}$	0.79 (0.67, 0.93)	0.006
IGP14	Continuous	1.41 (0.41)	1.49 (0.43)	0.66 (0.48, 0.89)	0.007	0.64 (0.47, 0.87)	0.004	0.73 (0.50, 1.06)	0.10
	RT			0.84 (0.74, 0.95)	0.005	0.83 (0.73, 0.94)	0.003	0.87 (0.75, 1.01)	0.07
IGP17	Continuous	7.99 (2.33)	8.33 (2.29)	0.93 (0.88, 0.99)	0.02	0.92 (0.87, 0.98)	0.007	0.95 (0.88, 1.02)	0.16
	RT			0.84 (0.74, 0.95)	0.007	0.81 (0.71, 0.93)	0.003	0.86 (0.73, 1.01)	0.07
IGP18	Continuous	1.88 (0.38)	1.89 (0.39)	0.89 (0.65, 1.23)	0.49	0.86 (0.63, 1.17)	0.34	0.94 (0.66, 1.33)	0.71
	RT			0.96 (0.85, 1.08)	0.47	0.95 (0.85, 1.07)	0.40	0.97 (0.85, 1.11)	0.69
**Sialylation**									
IGP24	Continuous	24.87 (3.25)	24.88 (3.03)	1.00 (0.96, 1.04)	0.93	0.99 (0.95, 1.03)	0.66	1.00 (0.96, 1.05)	0.85
	RT			0.98 (0.87, 1.12)	0.81	0.96 (0.85, 1.09)	0.56	1.00 (0.86, 1.16)	0.99
IGP25	Continuous	32.52 (6.35)	32.56 (6.35)	1.00 (0.98, 1.02)	0.98	1.00 (0.98, 1.02)	0.91	1.01 (0.98, 1.03)	0.62
	RT			1.00 (0.89, 1.13)	0.99	1.01 (0.90, 1.13)	0.90	1.04 (0.90, 1.19)	0.62
IGP26	Continuous	16.64 (3.67)	17.15 (3.58)	0.96 (0.93, 0.99)	0.02	0.95 (0.92, 0.99)	0.007	0.96 (0.92, 1.01)	0.11
	RT			0.85 (0.75, 0.97)	0.01	0.83 (0.73, 0.94)	0.004	0.86 (0.74, 1.01)	0.06

IGP27	Continuous	21.19 (5.12)	21.66 (4.95)	0.98 (0.96, 1.01)	0.17	0.98 (0.96, 1.01)	0.20	0.99 (0.96, 1.02)	0.63
	RT			0.91 (0.81, 1.03)	0.14	0.92 (0.82, 1.04)	0.20	0.96 (0.84, 1.11)	0.59
IGP29	Continuous	40.13 (2.97)	39.55 (2.71)	1.08 (1.03, 1.12)	0.001	1.06 (1.02, 1.10)	0.008	1.05 (1.00, 1.10)	0.05
	RT			1.24 (1.09, 1.41)	0.001	1.19 (1.05, 1.36)	0.007	1.17 (1.01, 1.36)	0.04
IGP31	Continuous	36.84 (3.79)	36.43 (3.97)	1.03 (1.00, 1.06)	0.10	1.03 (1.00, 1.06)	0.06	1.02 (0.98, 1.05)	0.32
	RT			1.11 (0.98, 1.26)	0.09	1.13 (1.00, 1.27)	0.05	1.08 (0.94, 1.24)	0.28
**Bisecting GlcNAc**									
IGP36	Continuous	0.29 (0.07)	0.29 (0.08)	4.00 (0.84, 19.03)	0.08	3.86 (0.75, 19.74)	0.11	4.17 (0.58, 29.87)	0.16
	RT			1.12 (0.99, 1.27)	0.06	1.12 (0.99, 1.27)	0.08	1.12 (0.96, 1.31)	0.14
IGP37	Continuous	0.17 (0.05)	0.16 (0.05)	6.17 (0.53, 72.39)	0.15	6.98 (0.55, 89.35)	0.14	7.93 (0.37, 168.5)	0.18
	RT			1.10 (0.97, 1.24)	0.13	1.11 (0.98, 1.26)	0.11	1.11 (0.95, 1.29)	0.19
IGP38	Continuous	0.14 (0.03)	0.14 (0.03)	17.82 (0.55, 577.2)	0.11	20.69 (0.56, 761.0)	0.10	21.73 (0.29, 1602)	0.16
	RT			1.11 (0.98, 1.26)	0.10	1.12 (0.98, 1.27)	0.09	1.12 (0.96, 1.30)	0.16
IGP39	Continuous	1.34 (0.33)	1.29 (0.30)	1.61 (1.10, 2.35)	0.02	1.52 (1.03, 2.25)	0.04	1.45 (0.90, 2.35)	0.13
	RT			1.16 (1.03, 1.32)	0.02	1.15 (1.01, 1.30)	0.03	1.11 (0.95, 1.30)	0.17
IGP40	Continuous	0.56 (0.06)	0.55 (0.06)	13.15 (1.49, 116.1)	0.02	10.21 (1.10, 95.12)	0.04	5.86 (0.39, 88.93)	0.20
	RT			1.16 (1.03, 1.31)	0.02	1.14 (1.01, 1.30)	0.04	1.11 (0.95, 1.30)	0.18
**Neutral IgG glycans**									
IGP43	Continuous	31.61 (7.90)	29.98 (7.18)	1.03 (1.01, 1.05)	0.0004	1.03 (1.02, 1.05)	0.0001	1.03 (1.01, 1.05)	0.004
	RT			1.24 (1.10, 1.41)	0.001	1.28 (1.12, 1.46)	0.0002	1.24 (1.06, 1.46)	0.008
IGP45	Continuous	7.73 (2.07)	7.31 (1.83)	1.11 (1.05, 1.18)	0.001	1.11 (1.04, 1.19)	0.001	1.08 (1.00, 1.17)	0.05
	RT			1.23 (1.08, 1.39)	0.001	1.22 (1.07, 1.40)	0.004	1.16 (0.98, 1.36)	0.08
IGP47	Continuous	22.73 (2.97)	23.17 (2.63)	0.94 (0.90, 0.99)	0.01	0.94 (0.90, 0.98)	0.009	0.94 (0.89, 1.00)	0.04
	RT			0.86 (0.75, 0.97)	0.02	0.85 (0.74, 0.97)	0.02	0.86 (0.74, 1.00)	0.06
IGP48	Continuous	11.72 (1.84)	12.15 (1.78)	0.88 (0.82, 0.94)	0.0002	0.88 (0.82, 0.94)	0.0003	0.86 (0.79, 0.94)	0.0004
	RT			0.79 (0.70, 0.90)	0.0002	0.79 (0.70, 0.90)	0.0003	0.76 (0.66, 0.89)	0.0004
IGP49	Continuous	6.86 (1.56)	6.85 (1.50)	1.00 (0.92, 1.08)	0.96	0.97 (0.89, 1.05)	0.47	0.96 (0.87, 1.06)	0.42
	RT			0.99 (0.87, 1.12)	0.88	0.95 (0.84, 1.07)	0.41	0.93 (0.81, 1.08)	0.36
IGP53	Continuous	13.10 (4.34)	14.10 (4.35)	0.94 (0.92, 0.97)	0.0002	0.94 (0.91, 0.97)	0.0001	0.95 (0.92, 0.99)	0.02
	RT			0.77 (0.68, 0.88)	$7.4x10^{-5}$	0.75 (0.65, 0.86)	$3.2x10^{-5}$	0.79 (0.67, 0.94)	0.007
IGP54	Continuous	1.76 (0.54)	1.87 (0.59)	0.72 (0.58, 0.91)	0.005	0.70 (0.56, 0.88)	0.003	0.78 (0.59, 1.04)	0.09
	RT			0.83 (0.73, 0.94)	0.004	0.82 (0.73, 0.93)	0.002	0.87 (0.74, 1.01)	0.07

**Galactosylation**									
IGP55	Continuous	40.35 (9.00)	38.27 (8.29)	1.03 (1.01, 1.04)	$8.3x10^{-5}$	1.03 (1.02, 1.05)	$2.4x10^{-5}$	1.03 (1.01, 1.05)	0.002
	RT			1.28 (1.12, 1.45)	0.0002	1.32 (1.15, 1.51)	$5.3x10^{-5}$	1.27 (1.08, 1.50)	0.005
IGP56	Continuous	43.11 (4.56)	43.99 (3.79)	0.95 (0.92, 0.98)	0.0003	0.94 (0.92, 0.97)	$6.8x10^{-5}$	0.94 (0.91, 0.97)	0.0005
	RT			0.82 (0.72, 0.93)	0.003	0.80 (0.70, 0.91)	0.001	0.79 (0.67, 0.93)	0.004
IGP57	Continuous	16.08 (5.03)	17.29 (5.12)	0.95 (0.93, 0.98)	0.0002	0.95 (0.92, 0.97)	0.0001	0.96 (0.93, 0.99)	0.02
	RT			0.77 (0.68, 0.88)	$6.2x10^{-5}$	0.75 (0.65, 0.86)	$2.9x10^{-5}$	0.80 (0.67, 0.94)	0.008
**Core fucosylation and bisecting GlcNAc**									
IGP62	Continuous	79.40 (3.90)	79.63 (3.54)	0.98 (0.95, 1.02)	0.34	0.99 (0.96, 1.03)	0.68	0.99 (0.95, 1.04)	0.77
	RT			0.95 (0.84, 1.08)	0.43	0.98 (0.87, 1.12)	0.80	0.99 (0.85, 1.15)	0.87
IGP63	Continuous	78.59 (4.39)	78.61 (4.18)	1.00 (0.97, 1.03)	0.99	1.01 (0.98, 1.04)	0.57	1.01 (0.97, 1.04)	0.67
	RT			1.01 (0.89, 1.14)	0.93	1.04 (0.92, 1.18)	0.51	1.04 (0.90, 1.21)	0.61
IGP64	Continuous	79.92 (3.92)	80.30 (3.65)	0.98 (0.94, 1.01)	0.14	0.98 (0.95, 1.02)	0.33	0.98 (0.95, 1.01)	0.43
	RT			0.92 (0.81, 1.04)	0.18	0.95 (0.84, 1.08)	0.41	0.95 (0.82, 1.10)	0.50
IGP66	Continuous	17.41 (3.29)	17.09 (2.98)	1.03 (0.99, 1.07)	0.12	1.02 (0.98, 1.06)	0.37	1.01 (0.96, 1.06)	0.68
	RT			1.09 (0.96, 1.23)	0.17	1.05 (0.92, 1.19)	0.49	1.02 (0.88, 1.19)	0.77
IGP67	Continuous	19.40 (3.95)	19.36 (3.72)	1.00 (0.97, 1.03)	0.94	0.99 (0.96, 1.02)	0.57	0.99 (0.95, 1.03)	0.62
	RT			1.00 (0.88, 1.13)	0.99	0.96 (0.85, 1.09)	0.51	0.96 (0.83, 1.11)	0.58
IGP68	Continuous	18.35 (3.54)	17.95 (3.34)	1.03 (1.00, 1.07)	0.08	1.02 (0.98, 1.06)	0.26	1.02 (0.97, 1.06)	0.44
	RT			1.10 (0.98, 1.25)	0.12	1.06 (0.94, 1.20)	0.34	1.05 (0.91, 1.22)	0.52
IGP70	Continuous	0.22 (0.05)	0.21 (0.05)	7.65 (0.66, 88.88)	0.10	3.42 (0.27, 42.66)	0.34	2.01 (0.10, 39.38)	0.65
	RT			1.09 (0.96, 1.23)	0.19	1.04 (0.92, 1.18)	0.53	1.02 (0.88, 1.19)	0.79
IGP71	Continuous	18.00 (3.49)	17.68 (3.15)	1.03 (0.99, 1.07)	0.14	1.02 (0.98, 1.06)	0.42	1.01 (0.96, 1.06)	0.68
	RT			1.08 (0.96, 1.23)	0.21	1.04 (0.92, 1.18)	0.55	1.03 (0.88, 1.19)	0.78
IGP72	Continuous	4.62 (1.06)	4.68 (1.04)	0.95 (0.84, 1.07)	0.40	0.99 (0.87, 1.12)	0.82	1.00 (0.86, 1.15)	0.95
	RT			0.94 (0.83, 1.06)	0.30	0.97 (0.86, 1.11)	0.69	0.99 (0.85, 1.15)	0.88

Supplementary Table 6 All-cause mortality analysis by stage [Model II; rank transformed variables]

Code	AJCC stage 1 (n=210)		AJCC stage 2 (n=327)		AJCC stage 3 (n=313)		AJCC stage 4 (n=102)	
	HR (95% CI)	p-value	HR (95% CI)	p-value	HR (95% CI)	p-value	HR (95% CI)	p-value
**Total IgG glycans (neutral and charged); Measured**								
IGP3	1.32 (0.96, 1.81)	0.09	1.93 (0.99, 3.78)	0.05	1.27 (1.05, 1.55)	0.02	1.15 (0.93, 1.42)	0.21
IGP5	1.29 (0.92, 1.82)	0.14	1.46 (1.16, 1.84)	0.001	1.20 (0.99, 1.47)	0.07	1.01 (0.83, 1.24)	0.90
IGP7	0.65 (0.47, 0.90)	0.009	0.76 (0.61, 0.96)	0.02	0.95 (0.79, 1.13)	0.55	1.00 (0.81, 1.22)	0.97
IGP8	0.77 (0.56, 1.06)	0.11	0.57 (0.46, 0.72)	9.3×10^{-7}	0.84 (0.71, 0.99)	0.04	1.25 (1.00, 1.57)	0.05
IGP9	0.95 (0.70, 1.29)	0.73	1.03 (0.83, 1.28)	0.77	0.98 (0.82, 1.18)	0.85	0.89 (0.70, 1.12)	0.32
IGP13	0.76 (0.55, 1.05)	0.09	0.72 (0.57, 0.93)	0.01	0.77 (0.62, 0.95)	0.02	0.91 (0.74, 1.12)	0.38
IGP14	0.89 (0.64, 1.23)	0.47	0.90 (0.72, 1.13)	0.38	0.85 (0.71, 1.03)	0.09	0.93 (0.73, 1.18)	0.55
IGP17	0.87 (0.63, 1.19)	0.38	0.79 (0.62, 1.01)	0.06	0.81 (0.67, 0.99)	0.04	0.79 (0.65, 0.97)	0.02
IGP18	0.91 (0.67, 1.23)	0.54	0.86 (0.70, 1.07)	0.17	1.02 (0.88, 1.19)	0.77	1.09 (0.85, 1.40)	0.49
**Sialylation**								
IGP24	1.19 (0.87, 1.64)	0.28	1.08 (0.86, 1.35)	0.51	0.92 (0.77, 1.09)	0.32	0.75 (0.63, 0.91)	0.003
IGP25	1.04 (0.76, 1.42)	0.80	0.97 (0.78, 1.20)	0.76	1.03 (0.88, 1.21)	0.70	1.00 (0.78, 1.28)	0.98
IGP26	0.90 (0.66, 1.22)	0.49	0.86 (0.68, 1.08)	0.20	0.80 (0.67, 0.97)	0.02	0.78 (0.63, 0.95)	0.01
IGP27	0.91 (0.66, 1.25)	0.57	0.84 (0.68, 1.03)	0.10	0.96 (0.82, 1.13)	0.66	0.98 (0.78, 1.23)	0.88
IGP29	1.43 (1.05, 1.96)	0.03	1.19 (0.95, 1.50)	0.13	1.11 (0.93, 1.33)	0.24	0.73 (0.60, 0.89)	0.002
IGP31	0.97 (0.71, 1.33)	0.84	0.87 (0.70, 1.07)	0.19	1.17 (0.99, 1.38)	0.06	1.39 (1.09, 1.76)	0.008
**Bisecting GlcNAc**								
IGP36	1.12 (0.81, 1.55)	0.48	1.18 (0.93, 1.49)	0.18	1.18 (0.98, 1.41)	0.08	1.24 (0.98, 1.57)	0.07
IGP37	1.04 (0.76, 1.42)	0.81	1.05 (0.85, 1.33)	0.70	1.19 (1.00, 1.43)	0.05	1.36 (1.09, 1.71)	0.008
IGP38	1.06 (0.77, 1.46)	0.72	1.06 (0.83, 1.35)	0.64	1.19 (1.00, 1.43)	0.05	1.36 (1.08, 1.71)	0.008
IGP39	1.17 (0.86, 1.59)	0.33	1.26 (1.00, 1.59)	0.05	1.23 (1.02, 1.47)	0.03	1.05 (0.86, 1.30)	0.63
IGP40	1.16 (0.85, 1.59)	0.34	1.25 (0.99, 1.58)	0.06	1.22 (1.02, 1.47)	0.03	1.05 (0.85, 1.29)	0.65
**Neutral IgG glycans**								
IGP43	1.35 (0.98, 1.85)	0.07	1.25 (0.98, 1.60)	0.07	1.27 (1.04, 1.54)	0.02	1.12 (0.91, 1.39)	0.29
IGP45	1.29 (0.92, 1.82)	0.14	1.49 (1.18, 1.88)	0.001	1.17 (0.96, 1.43)	0.11	0.96 (0.78, 1.19)	0.74
IGP47	0.67 (0.48, 0.92)	0.02	0.75 (0.60, 0.95)	0.02	0.89 (0.74, 1.07)	0.20	0.90 (0.74, 1.09)	0.29
IGP48	0.78 (0.57, 1.06)	0.12	0.57 (0.46, 0.72)	8.1×10^{-7}	0.79 (0.66, 0.95)	0.01	1.16 (0.92, 1.47)	0.20
IGP49	0.94 (0.68, 1.28)	0.68	1.01 (0.82, 1.25)	0.92	0.95 (0.80, 1.14)	0.58	0.83 (0.66, 1.05)	0.12

IGP53	0.77 (0.56, 1.06)	0.11	0.73 (0.57, 0.93)	0.01	0.77 (0.62, 0.94)	0.01	0.89 (0.72, 1.09)	0.25
IGP54	0.88 (0.64, 1.23)	0.46	0.90 (0.71, 1.12)	0.34	0.85 (0.70, 1.01)	0.07	0.90 (0.71, 1.12)	0.34
**Galactosylation**								
IGP55	1.39 (1.00, 1.92)	0.05	1.38 (1.08, 1.76)	0.009	1.31 (1.07, 1.60)	0.01	1.10 (0.89, 1.35)	0.38
IGP56	0.71 (0.51, 0.98)	0.04	0.69 (0.54, 0.87)	0.002	0.80 (0.66, 0.98)	0.03	0.95 (0.77, 1.17)	0.64
IGP57	0.78 (0.56, 1.07)	0.12	0.78 (0.61, 0.99)	0.05	0.76 (0.66, 0.93)	0.008	0.91 (0.73, 1.12)	0.35
**Core fucosylation and bisecting GlcNAc**								
IGP62	0.94 (0.68, 1.31)	0.73	0.75 (0.60, 0.94)	0.01	1.00 (0.84, 1.19)	0.99	1.03 (0.80, 1.32)	0.82
IGP63	1.03 (0.76, 1.41)	0.85	0.84 (0.64, 1.01)	0.06	1.03 (0.87, 1.23)	0.74	1.14 (0.91, 1.44)	0.25
IGP64	0.83 (0.60, 1.15)	0.26	0.77 (0.62, 0.96)	0.02	0.97 (0.81, 1.15)	0.69	1.09 (0.85, 1.41)	0.50
IGP66	1.13 (0.82, 1.55)	0.47	1.25 (1.00, 1.55)	0.05	1.03 (0.86, 1.23)	0.73	0.87 (0.70, 1.10)	0.24
IGP67	0.99 (0.73, 1.35)	0.97	1.15 (0.95, 1.49)	0.13	0.96 (0.80, 1.14)	0.64	0.84 (0.68, 1.05)	0.12
IGP68	1.20 (0.87, 1.66)	0.26	1.24 (1.00, 1.53)	0.05	1.05 (0.88, 1.25)	0.58	0.86 (0.68, 1.10)	0.23
IGP70	1.13 (0.81, 1.57)	0.47	1.28 (1.02, 1.59)	0.03	1.02 (0.86, 1.22)	0.79	0.88 (0.69, 1.11)	0.28
IGP71	1.11 (0.81, 1.53)	0.52	1.27 (1.02, 1.58)	0.03	1.02 (0.86, 1.22)	0.79	0.89 (0.71, 1.12)	0.32
IGP72	0.91 (0.66, 1.26)	0.59	0.79 (0.63, 0.98)	0.03	0.99 (0.83, 1.18)	0.92	1.11 (0.88, 1.40)	0.38

Supplementary Table 7 CRC-specific mortality analysis by stage [Model II; rank transformed variables]

Code	AJCC stage 1 (n=210)		AJCC stage 2 (n=327)		AJCC stage 3 (n=313)		AJCC stage 4 (n=102)	
	HR (95% CI)	p-value	HR (95% CI)	p-value	HR (95% CI)	p-value	HR (95% CI)	p-value
**Total IgG glycans (neutral and charged); Measured**								
IGP3	1.14 (0.67, 1.95)	0.63	1.21 (0.90, 1.62)	0.21	1.26 (1.02, 1.55)	0.04	1.15 (0.92, 1.43)	0.21
IGP5	0.94 (0.55, 1.61)	0.82	1.35 (1.01, 1.80)	0.04	1.10 (0.88, 1.36)	0.40	1.01 (0.82, 1.24)	0.94
IGP7	0.81 (0.48, 1.35)	0.42	0.74 (0.56, 0.98)	0.04	0.96 (0.79, 1.17)	0.71	1.00 (0.81, 1.24)	0.97
IGP8	0.72 (0.42, 1.24)	0.24	0.67 (0.51, 0.88)	0.004	0.85 (0.71, 1.02)	0.09	1.25 (0.99, 1.57)	0.06
IGP9	0.86 (0.52, 1.42)	0.56	1.00 (0.77, 1.31)	0.98	0.92 (0.76, 1.12)	0.42	0.88 (0.69, 1.12)	0.30
IGP13	0.98 (0.57, 1.69)	0.95	0.76 (0.56, 1.03)	0.08	0.78 (0.62, 0.98)	0.04	0.91 (0.71, 1.13)	0.39
IGP14	0.85 (0.50, 1.46)	0.56	0.91 (0.69, 1.20)	0.51	0.86 (0.70, 1.05)	0.13	0.92 (0.72, 1.18)	0.53
IGP17	1.12 (0.65, 1.94)	0.68	0.82 (0.61, 1.11)	0.20	0.85 (0.68, 1.05)	0.13	0.79 (0.64, 0.97)	0.02
IGP18	1.03 (0.62, 1.70)	0.92	0.84 (0.64, 1.08)	0.18	1.04 (0.88, 1.23)	0.64	1.07 (0.83, 1.38)	0.61
**Sialylation**								
IGP24	1.29 (0.77, 2.18)	0.34	1.05 (0.79, 1.39)	0.74	0.96 (0.79, 1.16)	0.64	0.75 (0.62, 0.90)	0.003
IGP25	1.23 (0.72, 2.11)	0.44	0.93 (0.71, 1.22)	0.61	1.08 (0.91, 1.29)	0.39	1.00 (0.78, 1.28)	0.99
IGP26	1.08 (0.64, 1.82)	0.77	0.87 (0.66, 1.16)	0.35	0.84 (0.68, 1.03)	0.09	0.77 (0.62, 0.95)	0.01
IGP27	1.19 (0.69, 2.05)	0.53	0.84 (0.64, 1.08)	0.18	1.02 (0.86, 1.22)	0.79	0.98 (0.77, 1.23)	0.85
IGP29	1.40 (0.85, 2.31)	0.19	1.17 (0.88, 1.55)	0.28	1.14 (0.94, 1.38)	0.20	0.73 (0.60, 0.90)	0.002
IGP31	1.05 (0.63, 1.73)	0.86	0.89 (0.69, 1.16)	0.41	1.18 (0.98, 1.42)	0.07	1.38 (1.08, 1.76)	0.01
**Bisecting GlcNAc**								
IGP36	1.02 (0.61, 1.72)	0.94	1.10 (0.82, 1.46)	0.53	1.15 (0.95, 1.40)	0.15	1.23 (0.97, 1.57)	0.09
IGP37	0.98 (0.59, 1.63)	0.94	0.99 (0.74, 1.33)	0.96	1.18 (0.97, 1.44)	0.10	1.36 (1.07, 1.71)	0.01
IGP38	0.99 (0.59, 1.65)	0.96	1.01 (0.75, 1.36)	0.93	1.18 (0.98, 1.44)	0.09	1.35 (1.07, 1.71)	0.01
IGP39	0.91 (0.55, 1.49)	0.71	1.22 (0.91, 1.63)	0.18	1.11 (0.91, 1.36)	0.29	1.05 (0.85, 1.30)	0.64
IGP40	0.91 (0.55, 1.50)	0.71	1.21 (0.90, 1.61)	0.21	1.11 (0.91, 1.36)	0.29	1.05 (0.85, 1.30)	0.66
**Neutral IgG glycans**								
IGP43	1.20 (0.70, 2.05)	0.51	1.21 (0.90, 1.63)	0.21	1.22 (1.02, 1.56)	0.03	1.12 (0.90, 1.40)	0.30
IGP45	0.95 (0.55, 1.63)	0.85	1.37 (1.02, 1.83)	0.03	1.08 (0.87, 1.33)	0.51	0.96 (0.77, 1.19)	0.69
IGP47	0.87 (0.51, 1.47)	0.60	0.74 (0.56, 0.99)	0.04	0.92 (0.75, 1.12)	0.41	0.90 (0.74, 1.10)	0.32
IGP48	0.78 (0.46, 1.31)	0.34	0.67 (0.51, 0.88)	0.003	0.82 (0.67, 0.99)	0.04	1.16 (0.91, 1.47)	0.23
IGP49	0.87 (0.53, 1.45)	0.60	0.99 (0.76, 1.29)	0.93	0.90 (0.74, 1.10)	0.30	0.82 (0.65, 1.04)	0.11

IGP53	1.00 (0.58, 1.72)	0.99	0.77 (0.57, 1.04)	0.09	0.79 (0.63, 0.98)	0.04	0.88 (0.72, 1.09)	0.26
IGP54	0.88 (0.51, 1.50)	0.63	0.91 (0.69, 1.20)	0.49	0.85 (0.70, 1.04)	0.12	0.89 (0.70, 1.12)	0.32
**Galactosylation**								
IGP55	1.14 (0.66, 1.97)	0.64	1.30 (0.96, 1.75)	0.09	1.27 (1.02, 1.59)	0.03	1.10 (0.89, 1.36)	0.40
IGP56	0.76 (0.45, 1.29)	0.32	0.73 (0.55, 0.98)	0.04	0.82 (0.66, 1.02)	0.07	0.95 (0.77, 1.18)	0.65
IGP57	0.96 (0.56, 1.65)	0.88	0.82 (0.61, 1.10)	0.18	0.78 (0.62, 0.97)	0.03	0.90 (0.73, 1.12)	0.36
**Core fucosylation and bisecting GlcNAc**								
IGP62	1.19 (0.69, 2.05)	0.53	0.80 (0.61, 1.05)	0.11	1.06 (0.88, 1.29)	0.52	1.04 (0.81, 1.33)	0.78
IGP63	1.23 (0.73, 2.06)	0.44	0.87 (0.65, 1.15)	0.33	1.10 (0.91, 1.33)	0.32	1.15 (0.91, 1.45)	0.25
IGP64	0.99 (0.58, 1.68)	0.96	0.80 (0.61, 1.06)	0.12	1.03 (0.85, 1.24)	0.77	1.11 (0.85, 1.43)	0.45
IGP66	0.88 (0.52, 1.47)	0.61	1.18 (0.90, 1.55)	0.22	0.96 (0.79, 1.17)	0.68	0.86 (0.69, 1.09)	0.21
IGP67	0.84 (0.50, 1.38)	0.49	1.14 (0.86, 1.50)	0.36	0.90 (0.74, 1.09)	0.29	0.84 (0.67, 1.04)	0.12
IGP68	0.98 (0.58, 1.64)	0.93	1.19 (0.91, 1.55)	0.19	0.98 (0.81, 1.19)	0.84	0.85 (0.67, 1.09)	0.20
IGP70	0.87 (0.51, 1.47)	0.61	1.22 (0.92, 1.60)	0.16	0.95 (0.78, 1.15)	0.61	0.87 (0.68, 1.10)	0.25
IGP71	0.87 (0.52, 1.46)	0.59	1.20 (0.92, 1.58)	0.18	0.95 (0.79, 1.16)	0.64	0.88 (0.70, 1.11)	0.29
IGP72	1.17 (0.70, 1.98)	0.55	0.83 (0.63, 1.09)	0.17	1.06 (0.88, 1.29)	0.54	1.12 (0.89, 1.42)	0.33

Supplementary Table 8 Multivariate Cox regression and estimate of the Harrell's concordance coefficient of the a) clinical parameters and b) clinical and glycan parameters by AJCC stage for all-cause mortality

	Stage 1		Stage 2		Stage 3		Stage 4	
**Clinical algorithm**	**All-cause mortality**		**All-cause mortality**		**All-cause mortality**		**All-cause mortality**	
	**HR (95% CI)**	**p-value**	**HR (95% CI)**	**p-value**	**HR (95% CI)**	**p-value**	**HR (95% CI)**	**p-value**
Age	1.07 (1.03, 1.11)	0.0006	1.04 (1.02, 1.07)	0.0009	1.02 (1.00, 1.04)	0.03	1.01 (0.99, 1.03)	0.44
Sex	0.73 (0.40, 1.34)	0.31	0.96 (0.63, 1.46)	0.85	0.95 (0.68, 1.33)	0.77	0.94 (0.63, 1.40)	0.76
CRP	2.84 (1.20, 6.78)	0.02	1.96 (1.06, 3.63)	0.18	1.70 (1.02, 2.83)	0.04	1.95 (1.18, 3.22)	0.01
BMI	1.02 (0.95, 1.10)	0.61	1.09 (1.04, 1.14)	0.0005	1.01 (0.97, 1.05)	0.49	1.04 (0.99, 1.08)	0.10
Harrell's C	*0.68*		*0.65*		*0.55*		*0.61*	
IDI	*n/a**		*0.09§*		*0.08§*		*0.13§*	
AUC	*n/a**		*0.66*		*0.58*		*0.63*	
**Clinical & glycans algorithm**	**All-cause mortality***		**All-cause mortality**		**All-cause mortality**		**All-cause mortality**	
	**HR (95% CI)**	**p-value**	**HR (95% CI)**	**p-value**	**HR (95% CI)**	**p-value**	**HR (95% CI)**	**p-value**
Age			1.04 (1.01, 1.06)	0.006	1.01 (0.99, 1.03)	0.23	1.00 (0.98, 1.02)	0.45
Sex			0.98 (0.64, 1.50)	0.93	0.94 (0.67, 1.32)	0.73	1.00 (0.66, 1.52)	0.67
CRP			2.33 (1.24, 4.39)	0.01	1.47 (0.88, 2.47)	0.14	1.53 (0.86, 2.73)	0.03
BMI			1.07 (1.02, 1.12)	0.003	1.01 (0.97, 1.05)	0.65	1.03 (0.98, 1.08)	0.05
Top Glycan 1**			0.59 (0.46, 0.76)	2.6×10^{-5}	1.06 (0.85,1.32)	0.61	1.11 (0.81, 1.51)	0.53
Top Glycan 2**			0.95 (0.77, 1.17)	0.63	1.39 (1.03, 1.89)	0.03	0.98 (0.72, 1.33)	0.90
Top Glycan 3**			1.06 (0.83, 1.35)	0.62	0.75 (0.55, 1.01)	0.06	0.77 (0.58, 1.02)	0.07
Harrell's C			*0.67*		*0.53*		*0.61*	
IDI			*0.15§§*		*0.12§§*		*0.41§§*	
AUC			0.72		*0.60*		*0.69*	

* Due to the low number of observations, cross-validation was not possible and therefore we could not calculate the IDI and AUC values. Harrell's C coefficient were calculated based on the fitting all dataset

§ The IDI was calculated based on the comparison of the model II (adjusted for AJCC, age and sex) and the full clinical model III (adjusted for stage, age, sex, BMI and CRP – presented here).

** Top Glycans for: Stage 2 IGP48, IGP18, IGP43; Stage 3 IGP43, IGP29, IGP24; Stage 4 IGP27, IGP49, IGP17

§§ The IDI was calculated based on the comparison of full clinical model III (adjusted for stage, age, sex, bmi and CRP) and the model with these clinical factors plus the three top selected glycans.

Supplementary Table 9 Multivariate Cox regression and estimate of the Harrell's concordance coefficient of the a) clinical parameters and b) clinical and glycan parameters by AJCC stage for CRC mortality

	Stage 1		Stage 2		Stage 3		Stage 4	
**Clinical algorithm**	**CRC mortality**		**CRC mortality**		**CRC mortality**		**CRC mortality**	
	**HR (95% CI)**	**p-value**	**HR (95% CI)**	**p-value**	**HR (95% CI)**	**p-value**	**HR (95% CI)**	**p-value**
Age	1.06 (1.00, 1.12)	0.05	1.03 (1.00, 1.06)	0.05	1.01 (0.99-1.03)	0.23	1.01 (0.99, 1.03)	0.48
Sex	0.59 (0.21, 1.64)	0.31	1.35 (0.82, 2.22)	0.24	1.02 (0.71-1.46)	0.92	0.97 (0.65, 1.44)	0.86
CRP	1.04 (0.93, 1.16)	0.49	2.09 (1.02, 4.3)	0.04	1.92 (1.13-3.25)	0.02	1.04 (0.99, 1.08)	0.09
BMI	2.52 (0.57, 11.19)	0.22	1.13 (1.07, 1.19)	7.9×10^{-6}	1.02 (0.98-1.06)	0.34	1.04 (0.99, 1.08)	0.07
Harrell's C	*0.68*		*0.63*		*0.55*		*0.56*	
IDI	*n/a**		*0.11*§		*0.06*§		*0.12*§	
AUC	*n/a**		*0.68*		*0.56*		*0.63*	
**Clinical & glycans algorithm**	**CRC mortality***		**CRC mortality**		**CRC mortality**		**CRC mortality**	
	**HR (95% CI)**	**p-value**	**HR (95% CI)**	**p-value**	**HR (95% CI)**	**p-value**	**HR (95% CI)**	**p-value**
Age			1.02 (0.99,1.05)	0.17	1.01 (0.99, 1.03)	0.22	1.01 (0.99, 1.03)	0.42
Sex			1.38 (0.83,2.27)	0.21	1.03 (0.71, 1.49)	0.87	1.09 (0.71, 1.66)	0.70
CRP			2.32 (1.10,4.87)	0.03	1.80 (1.06, 3.07)	0.03	2.22 (1.21, 4.06)	0.01
BMI			1.11 (1.05,1.17)	8.34E-05	1.02 (0.98, 1.06)	0.39	1.04 (0.99, 1.09)	0.12
Top Glycan 1**			0.81 (0.24,2.68)	0.73	0.91 (0.62, 1.34)	0.64	1.17 (0.87, 1.59)	0.29
Top Glycan 2**			0.87 (0.59,1.29)	0.50	1.15 (0.95, 1.40)	0.16	0.70 (0.55,0.90)	0.005
Top Glycan 3**			0.88 (0.29,2.63)	0.82	0.97 (0.66, 1.41)	0.87	0.87 (0.67, 1.13)	0.30
Harrell's C			*0.61*		*0.51*		*0.61*	
IDI			*0.14*§§		*0.08*§§		*0.43*§§	
AUC			*0.71*		*0.59*		*0.71*	

* Due to the low number of observations, cross-validation was not possible and therefore we could not calculate the IDI and AUC values. Harrell's C coefficient were calculated based on the fitting all dataset

§ The IDI was calculated based on the comparison of the model II (adjusted for AJCC, age and sex) and the full clinical model III (adjusted for stage, age, sex, BMI and CRP – presented here).

** Top Glycans for: Stage 2 IGP48, IGP56, IGP8; Stage 3 IGP67, IGP29, IGP49. IGP49, IGP63 and IGP9 were prioritised equally by generalised boosted approach, but results for IGP49 is only presented; Stage 4 IGP27, IGP24, IGP49

§§ The IDI was calculated based on the comparison of full clinical model III (adjusted for stage, age, sex, bmi and CRP) and the model with these clinical factors plus the three top selected glycans

Supplementary Table 10 Predictions of 5 year risk of CRC death for models with clinical factors and clinical and glycan factors using k-nearest neighbours, LASSO, Naïve Bayes, PAM, Support Vector Machines, Decision Trees, and Boosted Stump classifiers. The results are summarized over 10 cross-validation folds.

**Clinical model with age, sex and stage (n=950)**					
	**AUC**	**Accuracy**	**PPV**	**Sensitivity**	**Specificity**
Maximum Prior	0.5	0.7379	-	0	1
k Nearest Neighbours	0.6619	0.7284	0.4833	0.5221	0.8017
LASSO	0.7786	0.8189	0.8738	0.3614	0.9815
Naive Bayes normal	0.7627	0.8179	0.8654	0.3614	0.9800
Naive Bayes kernel	0.7587	0.8179	0.8654	0.3614	0.9800
PAM	0.7626	0.7379	-	0	1
SVM linear	0.6831	0.7253	0.4805	0.5944	0.7718
SVM quadratic	0.6854	0.7000	0.4503	0.6546	0.7161
SVM cubic	0.7047	0.6884	0.4434	0.7390	0.6705
SVM RBF	0.7014	0.7389	0.5016	0.6125	0.7803
Decision Trees	0.6430	0.8189	0.8738	0.3614	0.9815
Boosted stumps	0.7691	0.8200	0.8750	0.3655	0.9815
**Clinical model with age, sex, stage, BMI, CRP (n=950)**					
	**AUC**	**Accuracy**	**PPV**	**Sensitivity**	**Specificity**
Maximum Prior	0.5	0.7379	-	0	1
k Nearest Neighbours	0.6310	0.7211	0.4661	0.4418	0.8203
LASSO	0.8052	0.8105	0.7315	0.4378	0.9429
Naive Bayes normal	0.8076	0.8116	0.7108	0.4739	0.9315
Naive Bayes kernel	0.7962	0.8095	0.7931	0.3695	0.9658
PAM	0.8039	0.7379	-	0	1
SVM linear	0.7237	0.7337	0.4944	0.7028	0.7447
SVM quadratic	0.7096	0.7568	0.5315	0.6104	0.8088
SVM cubic	0.6884	0.7274	0.4840	0.6064	0.7703
SVM RBF	0.6965	0.7221	0.4776	0.6426	0.7504
Decision Trees	0.6476	0.8189	0.8738	0.3614	0.9815
Boosted stumps	0.7978	0.8189	0.7770	0.4337	0.9558

Note to **Supplementary Tables** 10-14: In cross-validation, because training sets overlap, prediction errors over different folds and samples are not independent, which significantly complicates estimation of distribution of prediction (generalization) errors[1]. Although it is not uncommon to report variances or confidence intervals of prediction errors, we find them misleading, as they ignore the covariance structure of the test errors across multiple folds. We refer the readers to the result from machine learning literature showing that there are no universal unbiased estimates of the variance of the cross-validation error, and that "naïve estimators that do not take into account the error correlations between training and test sets (can) grossly underestimate the variance"[2]. For some special cases there exist biased task- and model-specific estimates (for example, Markatou et. al.[3]), but to the best of our knowledge they have not yet been developed for the AUC criterion and our choice of the sparse parametric and non-parametric models chosen for their applicability to biomarker prediction studies. Supplementary Tables 10-16 summarize the aggregated AUCs over the

10 test folds not used during training, where all classifiers were calibrated to output conditional probabilities of the class given the covariates. Note that PPV, sensitivity, and specificity depend on the task-specific calibration; we show the results for the probability cut-off of 0.5.

Supplementary Table 11 Predictions of 5 year risk of CRC death for models with the extended set of clinical factors with and without glycans using k-nearest neighbours, LASSO, PAM, Support Vector Machines, Decision Trees, and Boosted Stump classifiers. The results are summarized over 10 cross-validation folds.

**Clinical model with age, sex, BMI, CRP, type of operation, time between operation and blood collection, and stage of cancer (n=949)**					
	**AUC**	**Accuracy**	**PPV**	**Sensitivity**	**Specificity**
Maximum Prior	0.5	0.7379	-	0	1
k Nearest Neighbours	0.6174	0.7081	0.4398	0.4274	0.8074
LASSO	0.8042	0.8061	0.7192	0.4234	0.9415
PAM	0.8036	0.7492	0.9167	0.0444	0.9986
SVM linear	0.7229	0.7292	0.4875	0.7097	0.7361
SVM cubic	0.7047	0.7408	0.5032	0.6290	0.7803
SVM RBF	0.6764	0.7144	0.4639	0.5968	0.7561
Decision Trees	0.6331	0.8188	0.8725	0.3589	0.9815
Boosted stumps	0.8086	0.8124	0.7431	0.4315	0.9472
**Clinical model with age, sex, BMI, CRP, type of operation, time between operation and blood collection, stage of cancer, and log-transformed glycans (n=949)**					
	**AUC**	**Accuracy**	**PPV**	**Sensitivity**	**Specificity**
Maximum Prior	0.5	0.7387	-	0	1
k Nearest Neighbours	0.5713	0.6881	0.3857	0.3266	0.8160
LASSO	0.7980	0.8093	0.7557	0.3992	0.9544
PAM	0.6918	0.7576	0.6667	0.1452	0.9743
SVM linear	0.7068	0.7208	0.4759	0.6774	0.7361
SVM cubic	0.6449	0.7218	0.4688	0.4839	0.8060
SVM RBF	0.5	0.7387	-	0	1
Decision Trees	0.6447	0.8188	0.8725	0.3589	0.9815
Boosted stumps	0.7849	0.8072	0.7305	0.4153	0.9458

Supplementary Table 12 Predictions of rapid progressors in stage 2 for models with the extended set of clinical factors with and without glycans using k-nearest neighbour, LASSO, PAM, Support Vector Machines, Decision Trees, and Boosted Stump classifiers. The results are summarized over 10 cross-validation folds.

**Clinical model for stage 2 with age, sex, BMI, CRP, type of operation, time between operation and blood collection, and stage of cancer (n=326)**					
	**AUC**	**Accuracy**	**PPV**	**Sensitivity**	**Specificity**
Maximum Prior	0.5	0.9356	-	0	1
k Nearest Neighbours	0.5863	0.8896	0.2000	0.2381	0.9344
LASSO	0.7820	0.9356	0.5000	0.0476	0.9967
PAM	0.7066	0.9356	-	0	1
SVM linear	0.7440	0.7699	0.1786	0.7143	0.7738
SVM quadratic	0.6939	0.8006	0.1765	0.5714	0.8164
SVM cubic	0.6200	0.8282	0.1569	0.3810	0.8590
SVM RBF	0.6331	0.8528	0.1860	0.3810	0.8852
Decision Trees	0.4746	0.9356	-	0	1
Boosted stumps	0.6911	0.9325	0.4286	0.1429	0.9869

**Clinical model for stage 2 with age, sex, BMI, CRP, type of operation, time between operation and blood collection, stage of cancer, and log-transformed glycans (n=326)**					
	**AUC**	**Accuracy**	**PPV**	**Sensitivity**	**Specificity**
Maximum Prior	0.5	0.9356	-	0	1
k Nearest Neighbours	0.5912	0.8988	0.2273	0.2381	0.9443
LASSO	0.7369	0.9356	0.5000	0.0476	0.9967
PAM	0.6623	0.9356	-	0	1
SVM linear	0.6537	0.7669	0.1429	0.5238	0.7836
SVM quadratic	0.5674	0.8957	0.1905	0.1905	0.9443
SVM cubic	0.4976	0.8896	0.0588	0.0476	0.9475
SVM RBF	0.5	0.9356	-	0	1
Decision Trees	0.4746	0.9356	-	0	1
Boosted stumps	0.5864	0.9294	0.2500	0.0476	0.9902

Supplementary Table 13 Predictions of rapid progressors in stage 3 for models with the extended set of clinical factors with and without glycans using k-nearest neighbour, LASSO, PAM, Support Vector Machines, Decision Trees, and Boosted Stump classifiers. The results are summarized over 10 cross-validation folds.

**Clinical model for stage 3 with age, sex, BMI, CRP, type of operation, time between operation and blood collection, and stage of cancer (n=312)**					
	**AUC**	**Accuracy**	**PPV**	**Sensitivity**	**Specificity**
Maximum Prior	0.5	0.8782	-	0	1
k Nearest Neighbours	0.4438	0.7596	0.0256	0.0263	0.8613
LASSO	0.6259	0.8782	-	0	1
PAM	0.4802	0.8782	-	0	1
SVM linear	0.5864	0.7115	0.1905	0.4211	0.7518
SVM quadratic	0.5412	0.6122	0.1453	0.4474	0.6350
SVM cubic	0.5133	0.6827	0.1325	0.2895	0.7372
SVM RBF	0.5360	0.6827	0.1494	0.3421	0.7299
Decision Trees	0.4901	0.8782	-	0	1
Boosted stumps	0.5965	0.8782	-	0	1
**Clinical model for stage 3 with age, sex, BMI, CRP, type of operation, time between operation and blood collection, stage of cancer, and log-transformed glycans (n=312)**					
	**AUC**	**Accuracy**	**PPV**	**Sensitivity**	**Specificity**
Maximum Prior	0.5	0.8782	-	0	1
k Nearest Neighbours	0.4972	0.7949	0.1176	0.1053	0.8905
LASSO	0.5953	0.8782	-	0	1
PAM	0.5645	0.8814	1	0.0263	1
SVM linear	0.5941	0.7051	0.1932	0.4474	0.7409
SVM quadratic	0.5169	0.7885	0.1500	0.1579	0.8759
SVM cubic	0.5107	0.8173	0.1481	0.1053	0.9161
SVM RBF	0.5000	0.8782	-	0	1
Decision Trees	0.4745	0.8782	-	0	1
Boosted stumps	0.4483	0.8494	0.2353	0.1053	0.9526

Supplementary Table 14 Predictions of rapid progressors in stage 4 for models with the extended set of clinical factors with and without glycans using k-nearest neighbour, LASSO, PAM, Support Vector Machines, Decision Trees, and Boosted Stump classifiers. The results are summarized over 10 cross-validation folds.

**Clinical model for stage 4 with age, sex, BMI, CRP, type of operation, time between operation and blood collection, and stage of cancer (n=102)**					
	**AUC**	**Accuracy**	**PPV**	**Sensitivity**	**Specificity**
Maximum Prior	0.5	0.7059	-	0	1
k Nearest Neighbours	0.5	0.5686	0.2941	0.3333	0.6667
LASSO	0.4331	0.6961	0.3333	0.0333	0.9722
PAM	0.4535	0.7059	-	0	1
SVM linear	0.5514	0.5588	0.3404	0.5333	0.5694
SVM quadratic	0.5819	0.6569	0.4138	0.4000	0.7639
SVM cubic	0.5472	0.6078	0.3529	0.4000	0.6944
SVM RBF	0.5278	0.6078	0.3333	0.3333	0.7222
Decision Trees	0.4899	0.7059	-	0	1
Boosted stumps	0.5505	0.6471	0.3125	0.1667	0.8472
**Clinical model for stage 4 with age, sex, BMI, CRP, type of operation, time between operation and blood collection, stage of cancer, and log-transformed glycans (n=102)**					
	**AUC**	**Accuracy**	**PPV**	**Sensitivity**	**Specificity**
Maximum Prior	0.5	0.7059	-	0	1
k Nearest Neighbours	0.5819	0.6569	0.4138	0.4000	0.7639
LASSO	0.5815	0.6863	0	0	0.9722
PAM	0.5257	0.6765	0	0	0.9583
SVM linear	0.5972	0.6373	0.4054	0.5000	0.6944
SVM quadratic	0.5778	0.6373	0.3939	0.4333	0.7222
SVM cubic	0.6486	0.7647	0.6875	0.3667	0.9306
SVM RBF	0.5000	0.7059	-	0	1
Decision Trees	0.6569	0.7353	0.6154	0.2667	0.9306
Boosted stumps	0.5565	0.6863	0.4500	0.3000	0.8472

Supplementary Table 15 Studies on IgG glycosylation changes in cancer

Author	Year	Cancer	Method	Samples	Cases	Controls	Results
**Galactosylation**							
Kanoh Y[4]	2004	Prostate cancer	Fluorophore-assocd. carbohydrate electrophoresis (FACE)	serum	12	10	Fr 1 (monogalactosyl oligosaccharide) and Fr 2 (digalactosyl oligosaccharide) decreased significantly ($p<0.05$), while Fr 4 (agalactosyl IgG oligosaccharide) increased with PCa tumor progression. The Fr 4 / Fr 1 + 2 ratio in metastatic PCa patients was significantly higher than in healthy controls ($p<0.05$)
Aurer I[5]	2007	Multiple myeloma	Lectin blotting and densitometry	blood	16	16	IgG galactosylation was reduced in multiple myeloma
Kodar K[6]	2011	Gastric cancer	LC-ESI-MS	serum	80	51	Significant increase of agalactosylated (GnGnF, GnGn(bi)F), and decrease of galactosylated (AGn(bi), AGn(bi)F, AA(bi), AAF)
Bones J[7]	2011	Gastric cancer	Hydrophilic interaction liquid chromatography with fluorescence detection	serum	80	30	The data indicates that in the cancerous state there is a switch in IgG production toward the more pro-inflammatory IgG G0 glycoform (agalactosyl).
Gercel-Taylor C[8]	2001	Ovarian cancer	Concanavalin A affinity columns and sodium dodecyl sulfate-polyacrylamide gel electrophoresis	serum	62	50	This report demonstrated the presence of an aberrantly glycosylated IgG population in cancer patients.
Saldova R[9]	2007	Ovarian cancer	Quantitative NPHPLC and exoglycosidase digestion	serum	27	34	IgG containing agalactosylated structures (G0) (mostly represented by FA2) were doubled; monogalactosylated (G1) decreased; digalactosylated (G2) structures decreased

Alley WR[10]	2012	Ovarian cancer	MALDI-TOF Mass-spectrometric Analysis	serum	19	20	Increased levels of a-galactosylation structures were obsd. on N-linked glycans derived from IgG, which were independent of the presence of fucose residues.
Qian Y[11]	2013	Ovarian cancer	MALDI-TOF Mass-spectrometric Analysis	serum	32	26	G0/(G1 + G2·2) was found significantly higher in the malignant group than in the benign group (0.74 vs 0.34; $p < 0.0001$)
**Sialylation**							
Flemming SC[12]	1998	Multiple myeloma	High pressure anion exchange chromatography with pulsed electrochemical detection (HPAE-PED)	serum	47	14	Patients with myeloma showed an increase in the proportion of sialylated oligosaccharides in comparison with patients with MGUS
Saldova R[9]	2007	Ovarian cancer	Quantitative NPHPLC and exoglycosidase digestion	serum	27	34	The overall sialylation decreased
Kodar K[6]	2011	Gastric cancer	LC-ESI-MS	serum	80	51	Decrease of monosialylated IgG glycoforms (NaAF, NaA(bi)) in cancer patients.
**Bisecting GlcNAC**							
Kodar K[6]	2011	Gastric cancer	LC-ESI-MS	serum	80	51	A statistically significant decrease of bisecting GlcNAc was observed in tumour stage II and III

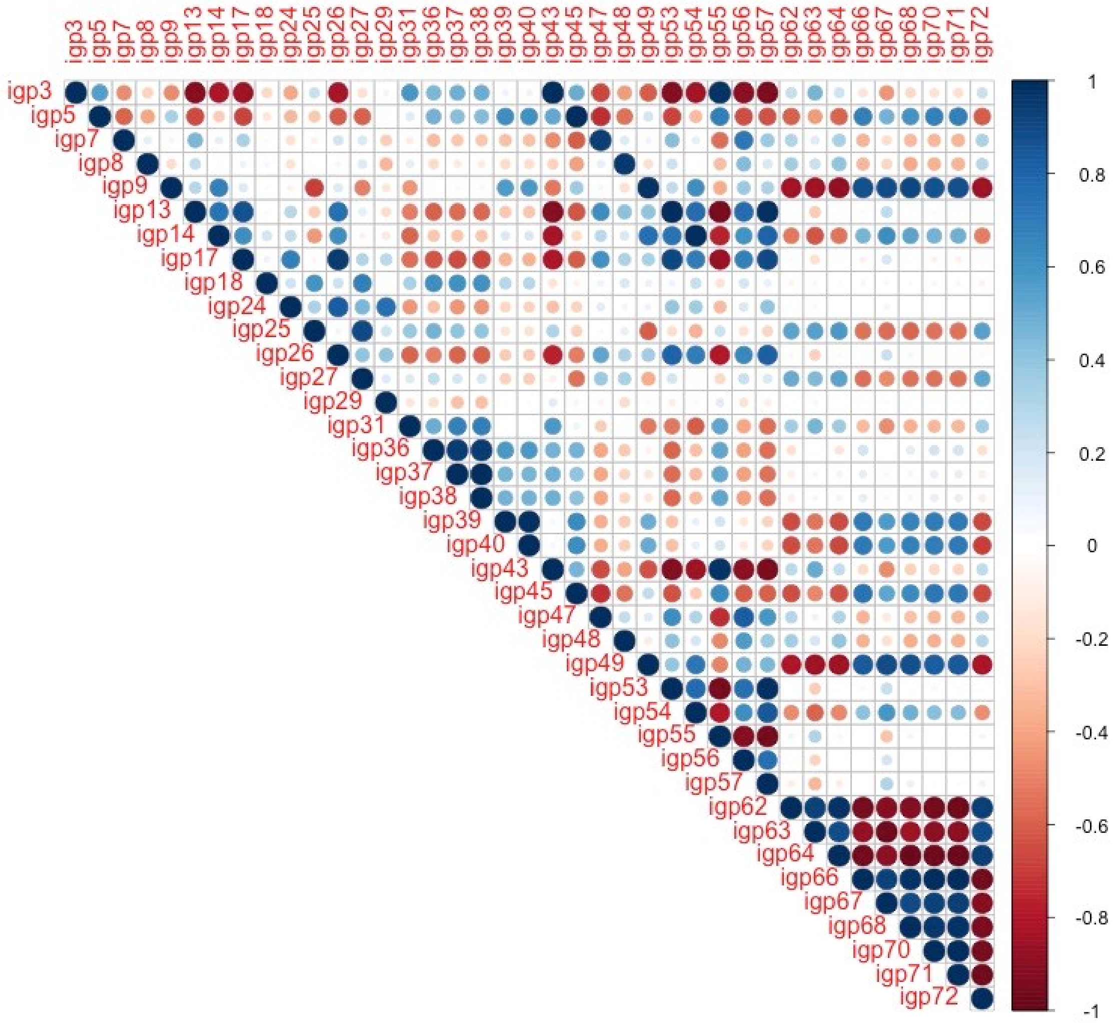

Supplementary Figure 1 Correlation matrix for robust measured and derived glycans

Supplementary Section: Model Selection

One classifier vs multiple classifiers

In life and clinical sciences, it is common to analyse the predictive performance by using an arbitrarily chosen single regression or classification model such as linear regression for continuous outcomes or logistic regression for binary outcomes, without motivating the model choice. There are multiple models that may in principle be considered for continuous and binary outcomes, and deeper insights about the utility of biomarkers may potentially be obtained by evaluating many such models. We note that the analysis of the predictive performance based on a single model may be misleading, due to the following observations: (i) It may happen that by considering a single model, researchers observe that biomarkers do not improve the quality of predictions. But this observation may be an artefact of the implied modelling constraints (such as the linear decision surface separating cases from controls in logistic regression). One reason for failing to demonstrate an improvement in predictions may be the fact that the chosen predictive model was limited and inappropriate for the dataset. The biomarkers may still be useful predictors, but the researchers may be making incorrect assumptions about the data and using a wrong model, without trying to evaluate whether the modelling assumptions are correct. (ii) A similar argument may hold for a subset of variables. For example, researchers may be able to demonstrate that a common model such as logistic regression with covariates defined by biomarkers and clinical variables outperforms logistic regression that only uses clinical variables, and may conclude that the biomarkers are generally useful for predicting the considered outcome. However, it may happen that the logistic assumption is particularly unfavourable to the clinical model (for example, when the mapping from the clinical variables to the outcomes is complex, and the classification surface cannot be well modelled by a hyperplane in the subspace of clinical variables). In this case, a clinical model of some other class (for example, an SVM) that does not use the biomarkers could significantly outperform models with biomarkers. In this case, the conclusion that the biomarkers are useful for developing a

diagnostics, may be misleading - one may be able to achieve a superior quality of predictions when considering "richer" clinical models (something overlooked by considering model of a single class).

We note that the assessment of the predictive performance by considering a single model may often be limited, and the results may need to be interpreted with some care. This work is an empirical attempt to overcome the arbitrariness of a specific model choice. In particular, we considered a larger set of models that make different assumptions about the mapping from glycans to outcomes. We use (nested) cross-validation to estimate the predictive performance on new previously unseen data. We then compare pairs of models of the same class that use clinical variables only and clinical variables with glycans, and test how likely it is that using glycans for predictions leads to improvements over clinical models independently of the modelling assumptions. (Note that the models are generally not nested even when they belong to the same class – so we cannot use standard tests). As the evaluation criterion, we use the AUC computed by cross-validation over the test folds of data. In some sense, rather than comparing an arbitrary model with or without glycans, we are evaluating how easy it may be to use glycomic biomarkers to construct a superior diagnostic independently of the modelling details.

Model Selection for Cross-Validation

We decided to approximate the performance on unused test data, where a possible chance improvement in the performance on training data is compensated by an implied penalty on the increased model complexity (Cherkassky and Mulier, 2007; Hastie, Tibshirani, and Friedman, 2009, see Chapter 7 in particular), because addition of uninformative covariates could have chance effects.

Cross-validation fits multiple models and tests them on non-overlapping sets of unused data. In contrast to fitting a single model to a complete dataset, this has the advantage of providing an approximately unbiased estimate of the expected performance in a new cohort, if the training and

validation samples are drawn from the same distribution (e.g. Hastie, Tibshirani, and Friedman (2009)). However, cross-validation has the disadvantage that there is no universal unbiased estimator of the variance of cross-validation estimates, which significantly complicates statistical testing (Bengio and Grandvalet, 2004), and which poses one of the big unsolved statistical problems in the area (Arlot and Celisse, 2010). This makes it important to choose an evaluation strategy: that is, to evaluate all models on the training dataset where statistical tests may be available, or to compute the expected performance on out-of-sample data – using cross-validation or similar evaluation techniques – at the expense of not being able to provide rigorous unbiased estimates of the error bars on the estimated performance. We insist that cross-validation is a general and appropriate strategy of assessing classifiers on new data, as the samples used for validations are independent from the training data, so at least the estimates of the mean performance are asymptotically unbiased (also see discussions in Hastie, Tibshirani, and Friedman (2009), Chapter 7, and Arlot and Celisse (2010), Section 4). However, despite its generality and wide use, some theoretical issues about cross-validation are still widely open (see e.g. Alrot and Celisse, 2010, Section 10). Demsar (2006) suggests Wilcoxon signed ranks or Friedman tests to compare multiple classifiers on large independent datasets; we use the former for comparing multiple models in the context discussed earlier.

Arlot and Celisse (2010). A survey of cross-validation procedures for model selection. Stat Surveys 4:40-79.

Bengio and Grandvalet (2004). No Unbiased Estimator of the Variance of K-Fold Cross-Validation. JMLR 5:1089-1105.

Cherkassky and Mulier (2007). Learning from Data (2nd ed.). Wiley.

Demsar (2006). Statistical Comparison of Classifiers over Multiple Datasets. JMLR 7:1-30.

Hastie, Tibshirani, Friedman (2009). The Elements of Statistical Learning (2nd ed.). Springer.

References

1. Arlot S, Celisse A. A survey of cross-validation procedures for model selection. *Statistics surveys* 2010;4:40-79.
2. Bengio Y, Grandvalet Y. No unbiased estimator of the variance of k-fold cross-validation. *Mach Learn Res* 2004;5:1089-105.
3. Markatou M, Tian H, Biswas S, Hripcsak GM. Analysis of variance of cross-validation estimators of the generalization error. *J Mach Learn Res* 2005;6:1127-68.
4. Kanoh Y, Mashiko T, Danbara M, Takayama Y, Ohtani S, Egawa S, et al. Changes in serum IgG oligosaccharide chains with prostate cancer progression. *Anticancer Res* 2004;24(5B):3135-9.
5. Aurer I, Lauc G, Dumic J, Rendic D, Matisic D, Milos M, et al. Aberrant glycosylation of Igg heavy chain in multiple myeloma. *Coll Antropol* 2007;31(1):247-51.
6. Kodar K, Stadlmann J, Klaamas K, Sergeyev B, Kurtenkov O. Immunoglobulin G Fc N-glycan profiling in patients with gastric cancer by LC-ESI-MS: relation to tumor progression and survival. *Glycoconj J* 2012;29(1):57-66.
7. Bones J, Byrne JC, O'Donoghue N, McManus C, Scaife C, Boissin H, et al. Glycomic and glycoproteomic analysis of serum from patients with stomach cancer reveals potential markers arising from host defense response mechanisms. *J Proteome Res* 2011;10(3):1246-65.
8. Gercel-Taylor C, Bazzett LB, Taylor DD. Presence of aberrant tumor-reactive immunoglobulins in the circulation of patients with ovarian cancer. *Gynecol Oncol* 2001;81(1):71-6.
9. Saldova R, Royle L, Radcliffe CM, Abd Hamid UM, Evans R, Arnold JN, et al. Ovarian cancer is associated with changes in glycosylation in both acute-phase proteins and IgG. *Glycobiology* 2007;17(12):1344-56.
10. Alley WR, Jr., Vasseur JA, Goetz JA, Svoboda M, Mann BF, Matei DE, et al. N-linked glycan structures and their expressions change in the blood sera of ovarian cancer patients. *J Proteome Res* 2012;11(4):2282-300.
11. Qian Y, Wang Y, Zhang X, Zhou L, Zhang Z, Xu J, et al. Quantitative analysis of serum IgG galactosylation assists differential diagnosis of ovarian cancer. *J Proteome Res* 2013;12(9):4046-55.
12. Fleming SC, Smith S, Knowles D, Skillen A, Self CH. Increased sialylation of oligosaccharides on IgG paraproteins--a potential new tumour marker in multiple myeloma. *J Clin Pathol* 1998;51(11):825-30.
